# Machine learning models for improving the diagnosing efficiency of skeletal class I and III in German orthodontic patients

**DOI:** 10.1038/s41598-025-97717-6

**Published:** 2025-04-13

**Authors:** Eva Paddenberg-Schubert, Kareem Midlej, Sebastian Krohn, Agnes Schröder, Obaida Awadi, Samir Masarwa, Iqbal M. Lone, Osayd Zohud, Christian Kirschneck, Nezar Watted, Peter Proff, Fuad A. Iraqi

**Affiliations:** 1 Department of Orthodontics, University Hospital of Regensburg, University of Regensburg, 93047 Regensburg, Germany; 2 Department of Clinical Microbiology and Immunology, Sackler Faculty of Medicine, Tel Aviv University, 6997801 Tel Aviv, Israel; 3 Center for Dentistry Research and Aesthetics, 4491800 Jatt, Israel; 4 Department of Orthodontics, University of Bonn, 53111 Bonn, Germany; 5 Department of Orthodontics, Faculty of Dentistry, Arab American University, Jenin, Palestine; 6 Gathering for Prosperity Initiative, 4491800 Jatt, Israel

**Keywords:** Class I, Class III, Malocclusion, Artificial intelligence, Orthodontic diagnostics, Individualized treatment planning, Diseases, Medical research

## Abstract

The precise and efficient diagnosis of an individual’s skeletal class is necessary in orthodontics to ensure correct and stable treatment planning. However, it is difficult to efficiently determine the true skeletal class due to several correlations between various anatomic structures. The primary outcome of this prospective cross-sectional study was developing a machine learning model for classifying patients as skeletal class I and III. Furthermore, the investigation intended to compare cephalometric variables between skeletal class I and III as well as between age and sex-specific subgroups to analyse correlations between cephalometric parameters and to perform Principal Component Analysis (PCA) to identify the most important variables contributing to skeletal class I and III variances. This study was based on the pre-treatment lateral cephalograms of 509 German orthodontic patients diagnosed as skeletal class I (n = 341) or III (n = 168) according to the individualised ANB of Panagiotidis and Witt, following descriptive analyses of cephalometric parameters, correlation analyses followed by Principal Component Analysis (PCA) to identify key cephalometric variables. Machine learning models, including Random Forest (RF), Classification and Regression Trees (CART), k-nearest Neighbors (KNN), Linear Discriminant Analysis (LDA), Support Vector Machines (SVM), and Generalized Linear Model (GLM), were evaluated for accuracy. Within the same skeletal class, age influenced cephalometric parameters: in skeletal class I, adolescents presented a more horizontal pattern (PFH/AFH, Gonial angle, NL-ML) and prominent mandible (SNB, SN-Pg) than children. In skeletal class III, the degree of sagittal discrepancy between jaw bases was most notable in adults (ANB: III_Age > 21-III _14 < Age < 20 − 1.78°). Comparing skeletal class I and III, the latter had more prognathic mandibles (SNB) and compensated incisors’ inclination (proclination of the upper (+ 1/NA: 9.01°), retroinclination of the lower incisors (− 1/ML: 8.99°). Among others, a correlation was found between the sagittal (degree of prognathism, SNB) and vertical (inclination, ML-NSL) orientation of the mandible (skeletal class I: p < 0.001, ρ = − 0.742; skeletal class III: p < 0.001, ρ = − 0.665). PCA revealed that the first four principal components explain 93% of the variance in skeletal class I/III diagnosis and that these parameters had the most influence loading score on the first component-PFH/AFH ratio (0.35), SNB angle (0.35), SN-Pg (0.37), and ML-NSL (− 0.35). Evaluating machine learning models, the general model, including all cephalometric parameters, age, and sex, resulted in perfect (1.00) accuracy and kappa scores compared to the gold standard Calculated_ANB with the model’s RF and CART. In model 2 the amount of input variables was reduced (Wits, SNB only), but the accuracy (0.88), and kappa (0.73) were still good in the KNN model. In the last section of this study, we applied different machine learning classification models. We examined the ability of the parameters—SNA, SNB, and ML-NSL angles to predict the classification as skeletal class I or III. The results demonstrated that the GLM model gained an accuracy of 0.99 (Accuracy = 0.99, Kappa = 0.97). The precise diagnosis of skeletal class I/III can be simplified by applying the machine learning model GLM with the input variables SNA, SNB, and ML-NSL only. This stresses the importance of their correct identification. However, considering all skeletal classes, a larger population is needed to validate and generalize this approach.

## Introduction

The correct diagnosis of an individual’s skeletal class, i.e., of the sagittal relation between the upper and the lower jaw, is mandatory in orthodontics to enable accurate and stable treatments in the long run. Determining the actual anteroposterior discrepancy is essential to differentiate between sagittal and vertical skeletal anomalies and, hence, to initiate the orthodontic treatment in the correct dimension. Skeletal class I presents the ideal anteroposterior relation between the upper and the lower jaw, while skeletal class III patients present a prognathic mandible and/ or retrognathic maxilla. This skeletal disharmony occurs in up to 26.7% of the population, although differences are observed between populations^1,2^.

Using lateral cephalograms, various methods exist to classify the skeletal class of patients. The angle ANB of Riedel^3^ is often used. Still, it does not account for the geometric and topographic relations between specific anatomic structures, and it was shown that the ANB angle depends on other parameters, requiring cautious interpretation^4^. Enlow described the interaction between various bony structures by the architectural equivalence between effective structures, which ideally results in balance during growth^5^. Solow identified associations between cephalometric parameters and explained the obligatory correlation between cephalometric parameters using the same reference point^6^. Thus, considering only the measured angle ANB may lead to false diagnoses of skeletal class, negatively affecting treatment planning. In contrast, the indiviudalised ANB angle introduced by Panagiotidis and Witt considers the inclination of the mandible and the degree of prognathism of the maxilla^7^. Using this method with floating norms, an ideal ANB angle can be calculated for each patient, which increases the precision in diagnosing the actual sagittal discrepancy. Such correlations exist for the ANB angle and other parameters like the Wits appraisal^8^ or the position of the lower incisors^9^. However, cephalometric correlations vary depending on the population^10,11^ and malocclusion^12^. Knowing the associations between variables allows for a better and individualised orthodontic treatment.

As various cephalometric parameters affect each other, it is necessary to identify the ones contributing the most to the parameter of interest to increase efficiency in the diagnostic process. This can be done by a Principal Component Analysis (PCA), which is intended to reduce the amount of input variables without losing significant information^13^. Applying this method in the skeletal class I/III diagnosis, the most relevant cephalometric parameters contributing to the skeletal class can be identified and considered during cephalometric analysis.

Artificial Intelligence (AI) has become increasingly important in dentistry, including orthodontics. Among others, it supports clinicians in orthodontic diagnostics, especially in cephalometric analysis and identification of reference landmarks^14–16^. It can also determine an individual’s skeletal class by applying machine learning models^17–19^. Various models can be used for this purpose, although, in general, none of them can be considered superior. Hence, the best-fitting model must be determined individually for each (research) question and population. Common machine learning models include Support Vector Machines (SVM), k-nearest neighbors (KNN), random forest (RF), Classification and Regression Tree (CART), linear discriminant analysis (LDA), and generalized linear model (GLM). The SVM method transforms given data points into vectors and generates a hyperplane to separate data points of different classes. Then, an unknown data point is classified based on its location in relation to the hyperplane. The KNN algorithm groups known data points according to their classification. The group of a new input variable is diagnosed based on the superior amount of neighboring, classified data points. In the technique RF, a new data point will be classified by the aggregated result of several randomly combined decision trees, which are largely independent. CART classifies new data points by using binary decision trees^20^. In LDA, the dimensions of a given data set are reduced, and regression between several input variables on the one hand and the dependent variable on the other hand, i.e., skeletal class I/III, is established to classify new data points. Finally, the GLM model is defined by three components: (1) a linear regression equation, (2) a specific error distribution, and (3) a link function, which is the transformation that links the predicted values for the dependent variable to the observed values^21^. GLM models extend linear mixed or hierarchical linear models to accommodate noncontinuous responses, such as binary responses or count^22^.

Recently, we investigated different machine learning models for diagnosing Arab orthodontic patients as skeletal class II or III^19^. However, due to the above-mentioned restrictions and the limited generalisability of a model, it cannot be applied to the distinction between skeletal class I and III in German orthodontic patients. Besides, the classical methods, like the individualised ANB angle introduced by Panagiotidis and Witt, and applied nowadays by orthodontists, do not fit all cases (r = 0.808)^7^. To our knowledge, no study has developed a machine learning model for skeletal class I/ III diagnosis in a German population. Hence, this multicentric, prospective cross-sectional study’s primary aim was to establish a machine learning model to correctly classify German orthodontic patients of all ages as skeletal class I or III. Secondary outcomes included comparing cephalometric parameters between groups, analyzing correlations between various variables, and using a PCA to identify the most important parameters for diagnosing skeletal class I and III.

## Material and methods

### Data collection

All methods were carried out in accordance with relevant guidelines and regulations. According to current guidelines and following the Ethics Committee of the University of Regensburg ethics and regulations, the committee reviewed and approved this research project and study design with approval number 19–1596-101 (dated 13/11/2019). Informed written consent was obtained from all participants. Besides, the orthodontists’ team collected all cephalometric data as codded records. The patients were coded according to their initials and serial numbers.

The study collective was comprised of orthodontic patients of any age and sex who were recruited at several orthodontic specialist offices in Germany and the Department of Orthodontics of the University Hospital of Regensburg, Germany. The pre-treatment lateral cephalograms of the participants, which had been taken for treatment purposes only, were analysed after anonymisation of the patients. To be included in the study, a pre-treatment lateral cephalogram was required as well as demographic information about age and sex. Only patients with skeletal class I or III, as diagnosed by the Calculated_ANB, based on the individualised ANB of Panagiotidis and Witt^7^, were included in this investigation.

- Calculated_ANB = ANB measured – ANB individual
- ANB individual = -35.16 + 0.4 × SNA + 0.2 × ML-NSL^7^
- Skeletal class I: -1.5° ≤ Calculated_ANB ≤ 1.5°
- Skeletal class III: Calculated_ANB < -1.5°

In this investigation, the limits applied for skeletal class I/ III deviated from those initially suggested by Panagiotidis and Witt^7^ to avoid the classification of borderline cases as skeletal class III. This modification allowed us to include the patients according to their actual diagnosis according to the orthodontist’s clinical diagnosis, as well as other vital parameters, like ANB angle and Wits appraisal.

Lateral cephalograms without the possibility of calibration or insufficient precision as well as patients presenting skeletal class II (Calculated_ANB > 1.5°) were excluded from the study. Finally, 509 patients were included in this investigation and retrospectively stratified into the groups skeletal class I (n = 341) and skeletal class III (n = 168).

### Sample size

The sample size was determined by the maximum number of cases available for the two skeletal classes of interest within the recruitment period. In addition, the machine learning models gained a powerful accuracy result based on the unseen (validation) data (n = 152).

Furthermore, patients were retrospectively allocated to age and sex-specific subgroups:

- Age: 0–13 years, 14–20 years, ≥ 21 years
- Sex: male, female
- Combination of sex and age subgroups

The analysis of the lateral cephalograms was performed digitally after calibration in the software Ivoris Analyze Pro, version 8.2.15.110 (Computer konkret AG, Falkenstein, Germany). All parameters evaluated are explained in Supplementary Figure S1 and Supplementary Table S1. Concerning the skeletal class, the gold standard used for determining an individual’s skeletal class was the method introduced by Panagiotidis and Witt^7^, whose formula is presented above.

Before the primary investigation, interrater and intrarater reliability of the cephalometric analysis was ensured by evaluating 50 randomly chosen lateral cephalograms twice by two different raters (SK, EP) as well as by the same rater with a time interval of at least two weeks in between. The results of the cephalometric analysis were descriptively analysed and compared between skeletal class I and III, as well as between subgroups of age and sex, using Tukey correction for multiple comparisons. Then, Spearman correlations between cephalometric parameters were evaluated, followed by the PCA.

### Data normality

In this study, we assumed normal distribution using the central limit theorem (CLT), which suggests that the data tends to be approximately normal if the sample size exceeds 30. According to the CLT, the sampling distribution approximates the standard normal distribution if the sample size is 30 ^23^. However, for the ANOVA tests and correlation comparisons, we grouped our study participants into subgroups of sex and age within each class. We received less than 30 participants in the following subgroups—skeletal Class I: males and females older than 21 and males aged between 14 and 20 years. And among skeletal class III patients: adolescents and old males and females (i.e., Age > 14). For these groups, we conducted Shapiro–Wilk tests that showed a normal distribution in the majority (152 out of 168) of the parameters in all subgroups. The results of the Shapiro–Wilk test are presented in full detail in Supplementary table S2.”

### Data balancing

This study included patients with skeletal class I (n = 341) and skeletal class III (n = 168). Therefore, machine learning models might be influenced by these imbalanced groups. To deal with this problem, we performed the same analysis on the original data and then repeated the models after downsampling the groups, using the R caret function – downsample (). The balanced groups contained 118 patients in each group.^24,25^.

### Machine learning models

We preprocessed the data for each machine learning model through centering and scaling functions to improve the model’s performance. For this study’s primary outcome, i.e., the establishment of a machine learning model for diagnosing skeletal class I and III, different machine learning models were tested concerning the accuracy, reliability (kappa), sensitivity, and specificity compared to the gold standard Calculated_ANB^7^. The machine learning models varied regarding the number of input variables and the model type. The following models were tested to find the best-fitting one: Support Vector Machine (SVM), K-Nearest Neighbors (KNN), Random Forest (RF), Classification and Regression Tree (CART), Linear Discriminant Analysis (LDA), and generalized linear model (GLM). The number of input variables was reduced based on the importance of each parameter contributing to the diagnosis of skeletal class I/III in the general machine learning model. In models 1 to 3, only the most important variables with decreasing relevance were considered input variables. Besides, we applied the same machine learning classification models to examine the ability of the parameters that define ANB angle (i.e., SNA-SNB) and ANBind (i.e., the equation ANBind = − 35.16 + 0.4 × SNA + 0.2 × ML-NSL^7^), the parameters- SNA, SNB, and ML-NSL angles to predict the skeletal classification as class I or III. Finally, the best fitting of each model was validated using statistics (sensitivity, specificity) and graphical illustration (confusion matrix).

### Statistical analysis

First, the interrater and intrarater reliability of the cephalometric analyses were tested using the test–retest method. Statistical analysis was done with the R software platform (https://www.r-project.org/). One-way analysis of variance (ANOVA) was performed to analyse differences in cephalometric parameters between skeletal classes I and III. Then, post-hoc Tukey analyses were conducted to investigate differences and, hence, the effect of age and sex on cephalometric variables within the same and between different skeletal classes. Afterward, Spearman correlation was calculated to identify correlations between specific cephalometric variables and illustrated via Heatmap correlation matrices. The limits of Cohen^26^ were applied to interpret the degree of correlation: |ρ|≤ 0.1 was regarded as a weak correlation, |ρ|≤ 0.3 as a moderate, and |ρ|> 0.5 as a strong correlation. This analysis was done for each skeletal class and age and sex-specific subgroups. The significance and high significance levels were set at p < 0.05 and p < 0.01, respectively. Regarding PCA, the percentage of variance in skeletal class I/III diagnosis explained by the principal components was given after data had been normalized. Furthermore, the cosine squared function computed and visualized the loading values of all cephalometric parameters on the first four principal components. All machine learning classifications were done using the caret function. The caret package (short for classification and regression training) contains functions to streamline the model training process for complex regression and classification problems^24,25^. The performance of machine learning models was first tested by evaluating their accuracy and reliability (kappa) derived from tenfold cross-validation.

### Validation process

Initially, we divided the data into 70% for training and 30% for validation. Then, we used the training data with a k-fold cross-validation. The k-fold cross validation is a common procedure for estimating the performance of a classification algorithm. In this process, we randomly divide the data set into k disjoint folds with approximately equal size, and each fold is used to test the model produced from the k-1 folds. In the next step, we evaluate the average of the k accuracies resulting from this process^27^. In this research, we used tenfold cross-validation defined by the R packages Caret, and it was used for all models to evaluate their performances (i.e., mean accuracy). In other words, for each model, the data set was divided to train in 9 splits and test on 1 split and then iterated through all combinations of train-test splits. Finally, the best fitting model, which was assessed using the mean accuracy, was validated using the independent validation set, which included 30% of the data, by comparing the skeletal class diagnosis obtained by the gold standard Calculated_ANB with the machine learning model and calculating sensitivity and specificity.

## Results

### Demographic data

Among the 509 patients included, 341 (67%) presented skeletal class I and 168 skeletal class III (33%). For both skeletal classes, a similar distribution concerning age and sex was observed, but children (aged 0–13 years) were the dominant subgroup (Table 1).

**Table 1:** Demographic data of the study collective.

Group	n	Age				Age subgroups (years, %)			Sex	
		Mean	SD	min	max	0–13	14–20	> 21	Male	Female
Skeletal class I	341	13	4	6.6	41	270, 79%	57, 17%	14, 4%	148, 43%	193, 57%
Skeletal class III	168	14	6.2	5.3	49	119, 71%	31, 18%	18, 11%	76, 45%	92, 55%

*SD* standard deviation, *min* minimum, *max* maximum, *n* absolute numbers, *%* relative frequency.

### Cephalometric parameters

Interrater (0.92 to 0.99) and intrarater reliability (0.90 to 0.99) were almost perfect, ensuring reproducible cephalometric measurements.

The descriptive data of the cephalometric analysis, separated for skeletal class I and III, is reported in Supplementary Material Table S3. Table 2 presents the significant differences between various age and sex-specific subgroups separately for each skeletal class. Among skeletal class I patients, adolescents (age 14–20) had a more horizontal growth pattern (PFH/AFH ratio: + 2.14°, Gonial angle: -2.73°) and hypodivergent jaw bases (NL-ML: -2.23°) than children (age 0–13 years). Moreover, in adolescents, the mandible (SNB: + 1.16°) and chin (SN-Pg: + 1.33°) were more prognathic than in children. Concerning dental parameters, males presented higher proclination of the upper incisors than females (+ 1/NSL: − 1.96°, + 1/NA: 1.83°). In skeletal class III, the sagittal discrepancy between the upper and lower jaw was more pronounced in adults than in growing patients, especially in males (ANB: III _Age > 21-III _14 < Age < 20 − 1.78°).

**Table 2:** Significant differences in cephalometric parameters between age and sex specific subgroups within each skeletal class.

Parameter	Group A_Group B	Difference	Lower CI	Upper CI	Tukey Adj. P value
Skeletal class I					
PFH/AFH ratio [%]	I_14 < Age < 20-I_0 < Age < 13	2.14	0.00	4.28	0.05
Gonial angle [°]	I_14 < Age < 20-I_0 < Age < 13	− 2.73	− 5.23	− 0.22	0.02
S–N (mm)	I_Male-I_Female	2.00	0.80	3.20	0.00
S–N (mm)	I_14 < Age < 20-I_0 < Age < 13	1.98	0.21	3.76	0.02
S–N (mm)	I_Male_0 < Age < 13-I_Female_0 < Age < 13	1.99	0.31	3.66	0.01
S–N (mm)	I_Male_14 < Age < 20-I_Female_0 < Age < 13	4.47	1.53	7.41	0.00
Go-Me [mm]	I_Male-I_Female	1.87	0.31	3.44	0.01
Go-Me [mm]	I_14 < Age < 20-I_0 < Age < 13	4.87	2.70	7.04	0.00
Go-Me [mm]	I_Female_14 < Age < 20-I_Female_0 < Age < 13	4.41	1.14	7.69	0.00
Go-Me [mm]	I_Male_14 < Age < 20-I_Female_0 < Age < 13	7.37	3.73	11.00	0.00
Go-Me [mm]	I_Male_14 < Age < 20-I_Male_0 < Age < 13	5.43	1.72	9.14	0.00
+ 1/NA [°]	I_14 < Age < 20-I_0 < Age < 13	− 3.47	− 6.60	− 0.33	0.02
+ 1/NA [°]	I_Male_0 < Age < 13-I_Female_14 < Age < 20	4.99	0.08	9.90	0.04
NL-ML [°]	I_14 < Age < 20-I_0 < Age < 13	− 2.23	− 4.19	− 0.26	0.02
Gonial angle [°]	I_Male_14 < Age < 20-I_Male_0 < Age < 13	− 3.65	− 7.28	− 0.01	0.05
SNB [°]	I_14 < Age < 20-I_0 < Age < 13	1.16	0.08	2.25	0.03
SN-Pg [°]	I_14 < Age < 20-I_0 < Age < 13	1.33	0.21	2.44	0.02
Go-Me [mm]	I_Male_0 < Age < 13-I_Female_0 < Age < 13	1.94	0.21	3.67	0.02
Go-Me [mm]	I_Male_Age > 21-I_Male_14 < Age < 20	− 7.58	− 13.99	− 1.17	0.01
Wits appraisal [mm]	I_Male-I_Female	0.62	0.14	1.10	0.01
Wits appraisal [mm]	I_Male_14 < Age < 20-I_Female_0 < Age < 13	1.46	0.07	2.85	0.03
+ 1/NSL [°]	I_Male-I_Female	− 1.96	− 3.77	− 0.15	0.03
+ 1/NA [°]	I_Male-I_Female	1.83	0.12	3.53	0.04
+ 1/NA [mm]	I_Age > 21-I_14 < Age < 20	1.82	0.01	3.64	0.05
Skeletal class III					
ANB [°]	III_Age > 21-III_0 < Age < 13	− 1.68	− 3.01	− 0.36	0.00
ANB [°]	III_Age > 21-III_14 < Age < 20	− 1.78	− 3.33	− 0.23	0.01
ANB [°]	III_Male_Age > 21-III_Female_0 < Age < 13	− 2.55	− 4.79	− 0.30	0.01
S–N (mm)	III_Male-III_Female	1.86	0.16	3.55	0.03
S–N (mm)	III_14 < Age < 20-III_0 < Age < 13	3.02	0.56	5.48	0.01
S–N (mm)	III_Female_14 < Age < 20-III_Female_0 < Age < 13	4.46	0.34	8.58	0.02
S–N (mm)	III_Male_0 < Age < 13-III_Female_0 < Age < 13	2.39	0.22	4.57	0.02
S–N (mm)	III_Male_14 < Age < 20-III_Female_0 < Age < 13	3.71	0.10	7.33	0.04
Go-Me (mm)	III_14 < Age < 20-III_0 < Age < 13	5.30	2.29	8.30	0.00
Go-Me (mm)	III_Age > 21-III_0 < Age < 13	6.87	3.11	10.64	0.00
Go-Me [mm]	III_Female_14 < Age < 20-III_Female_0 < Age < 13	5.15	0.05	10.24	0.05
Go-Me [mm]	III_Male_14 < Age < 20-III_Female_0 < Age < 13	5.99	1.54	10.45	0.00
Go-Me [mm]	III_Male_Age > 21-III_Female_0 < Age < 13	9.93	3.63	16.22	0.00
Go-Me [mm]	III_Male_14 < Age < 20-III_Male_0 < Age < 13	5.18	0.55	9.81	0.01
Go-Me [mm]	III_Male_Age > 21-III_Male_0 < Age < 13	9.11	2.70	15.53	0.00
Calculated_ANB [°]	III_Age > 21-III_0 < Age < 13	− 1.28	− 2.13	− 0.44	0.00
Calculated_ANB [°]	III_Age > 21-III_14 < Age < 20	− 1.37	− 2.35	− 0.38	0.00
Calculated_ANB [°]	III_Male_Age > 21-III_Female_0 < Age < 13	− 1.55	− 2.98	− 0.12	0.02
Calculated_ANB [°]	III_Male_Age > 21-III_Female_14 < Age < 20	− 1.92	− 3.64	− 0.20	0.01
+ 1/NA [°]	III_Male-III_Female	2.26	0.22	4.30	0.03
+ 1/NA [mm]	III_Male-III_Female	0.85	0.10	1.61	0.03

*CI* confidence interval, *Tukey Adj. P value* Tukey adjusted p-value.

The significant differences between age and sex-specific subgroups across skeletal class I and III are presented in Supplementary Material Table S4. Whereas the maxillary prognathism was not different between skeletal class I and III (SNA), the mandible was more prognathic in skeletal class III compared to skeletal class I (SNB). This observation was pronounced if class III adults were included in the comparison (III_Age > 21-I_0 < Age < 13: SNB-difference 4.06°). In the vertical direction, the growth pattern was more horizontal in skeletal class III according to facial axis (III_Female_14 < Age < 20-I_Female_0 < Age < 13: difference 4.33°), but more vertical in skeletal class III according to Gonial angle (III_Male_0 < Age < 13-I_Male_14 < Age < 20: difference 6.86°). However, this observation was not significant in adult patients (age > 21). The upper incisors were more proclined (+ 1/NL, + 1/NSL, + 1/NA) and anteriorly positioned (+ 1/NA [mm]) in skeletal class III than in skeletal class I (e.g., + 1/NA [°]: III_Male_0 < Age < 13-I_Female_14 < Age < 20, difference: 9.01°), whereas the lower incisors were more retroinclined in skeletal class III (-1/ML: III _Age > 21-I _Age > 21, difference: 8.99°).

### Spearman correlation between cephalometric measurements

**Fig. 1:**
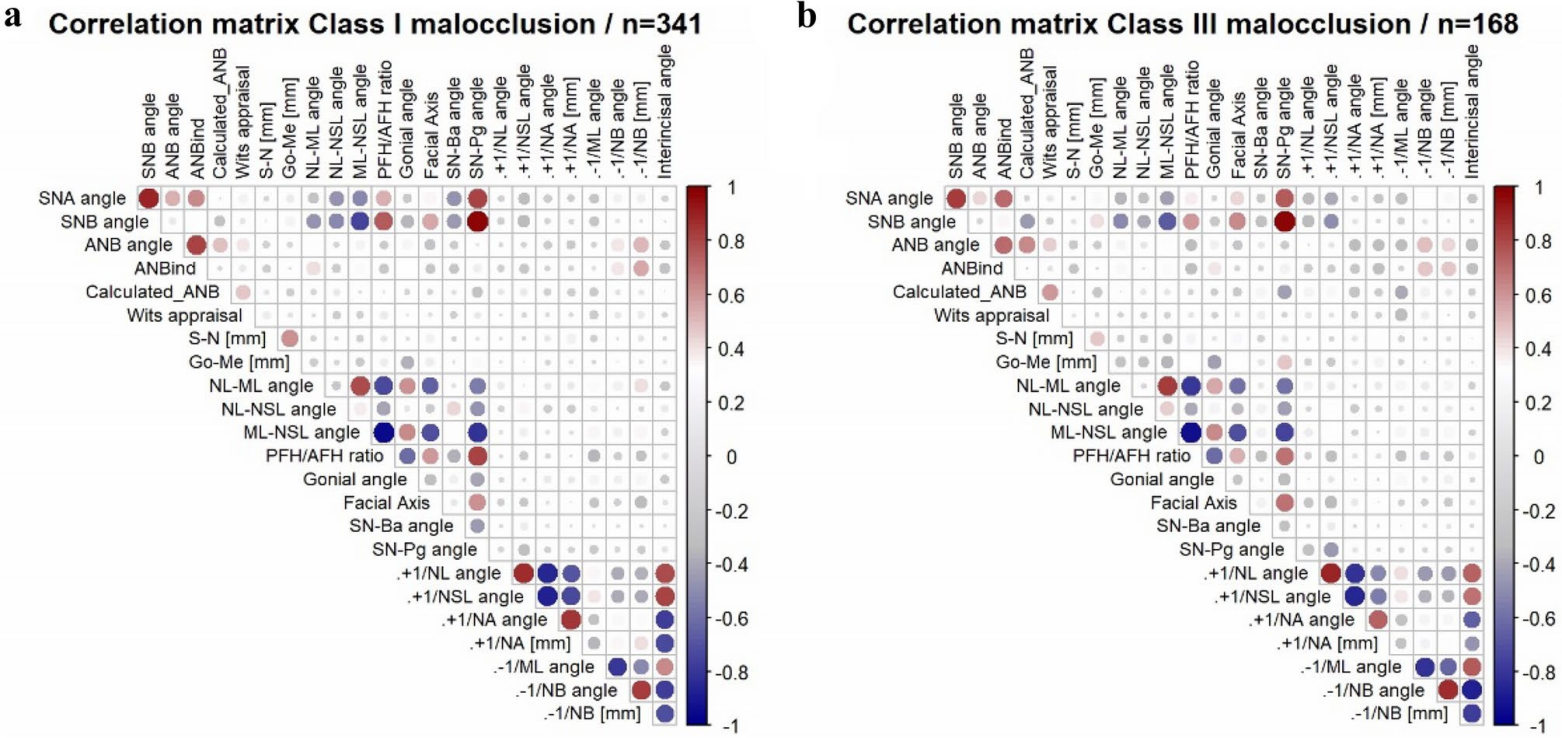
Heatmap correlation matrix showing the correlations between cephalometric parameters for patients with skeletal class I (1a) and III (1b).

The results of the correlation analyses between various cephalometric parameters within each skeletal class are presented in Fig. 1a,b. Generally, similar correlations were observed for both skeletal classes. In the vertical direction, there was a strong negative association between the growth pattern PFH/AFH and the inclination of the mandible ML/NSL in class I (p < 0.001, ρ = -0.956) and III (p < 0.001, ρ = 0-0.934). In the sagittal direction, a strong positive correlation was found between the sagittal position of the chin (SN-Pg) and the degree of mandibular prognathism (SNB) in class I (p < 0.001, ρ = 0.964) and III (p < 0.001, ρ = 0.962). Furthermore, the degree of maxillary prognathism (SNA) was strongly positively related to the mandibular one (SNB) in both classes (skeletal class I: p < 0.001, ρ = 0.896; skeletal class III: p < 0.001, ρ = 0.827). Also, a strong negative correlation between the sagittal and vertical direction was observed, for example, for the mandible’s degree of prognathism (SNB) and its inclination (ML-NSL) (skeletal class I: p < 0.001, ρ = -0.742; skeletal class III: p < 0.001, ρ = -0.665). Moreover, in both skeletal classes, strong correlations between dental parameters, which measured the inclination of the same teeth to various reference lines, were found (e.g., + 1/SNL and + 1/NL angle in skeletal class I: p < 0.001, ρ = -0.879).

Concerning Calculated_ANB and thereby the diagnosis of skeletal class, various significant correlations were found. In both skeletal classes, moderate correlations of Calculated_ANB were found with the mandible’s position(SNB—skeletal class I: p < 0.001, ρ = -0.262; skeletal class III: p < 0.001, ρ = -0.459) and chin’s sagittal position (SN-Pg—skeletal class I: p < 0.001; ρ = -0.240; skeletal class III: p < 0.001, ρ = 0.428) and mandibular length (skeletal class I – p < 0.01, ρ = -0.151; skeletal class III: p < 0.01 , ρ = 0.214). Additionally, in skeletal class III, a moderate correlation existed between Calculated_ANB and the Facial axis (p = 0.019, ρ = -0.180), but in skeletal class I only, a moderate correlation was identified between Calculated_ANB and the maxilla’s inclination (NL-NSL; p = 0.03, ρ = -0.112). Whereas the correlation between Calculated_ANB on the one hand and ANB angle (p < 0.001, ρ = 0.495) and Wits appraisal (p < 0.001, ρ = 0.474) on the other hand was moderate in skeletal class I, it was identified to be strong in skeletal class III (ANB: p < 0.001, ρ = 0.621; Wits appraisal: p < 0.001, ρ = 0.598).

The heatmap correlation matrices of age and sex-specific subgroups are presented in the Supplementary Material Figures S2i-vi and S3i-vi. Generally, the number of significant correlations increased at higher ages for both classes. In skeletal class III, male adults had more significant associations than females.

### Principal component analysis (PCA)

**Fig. 2:**
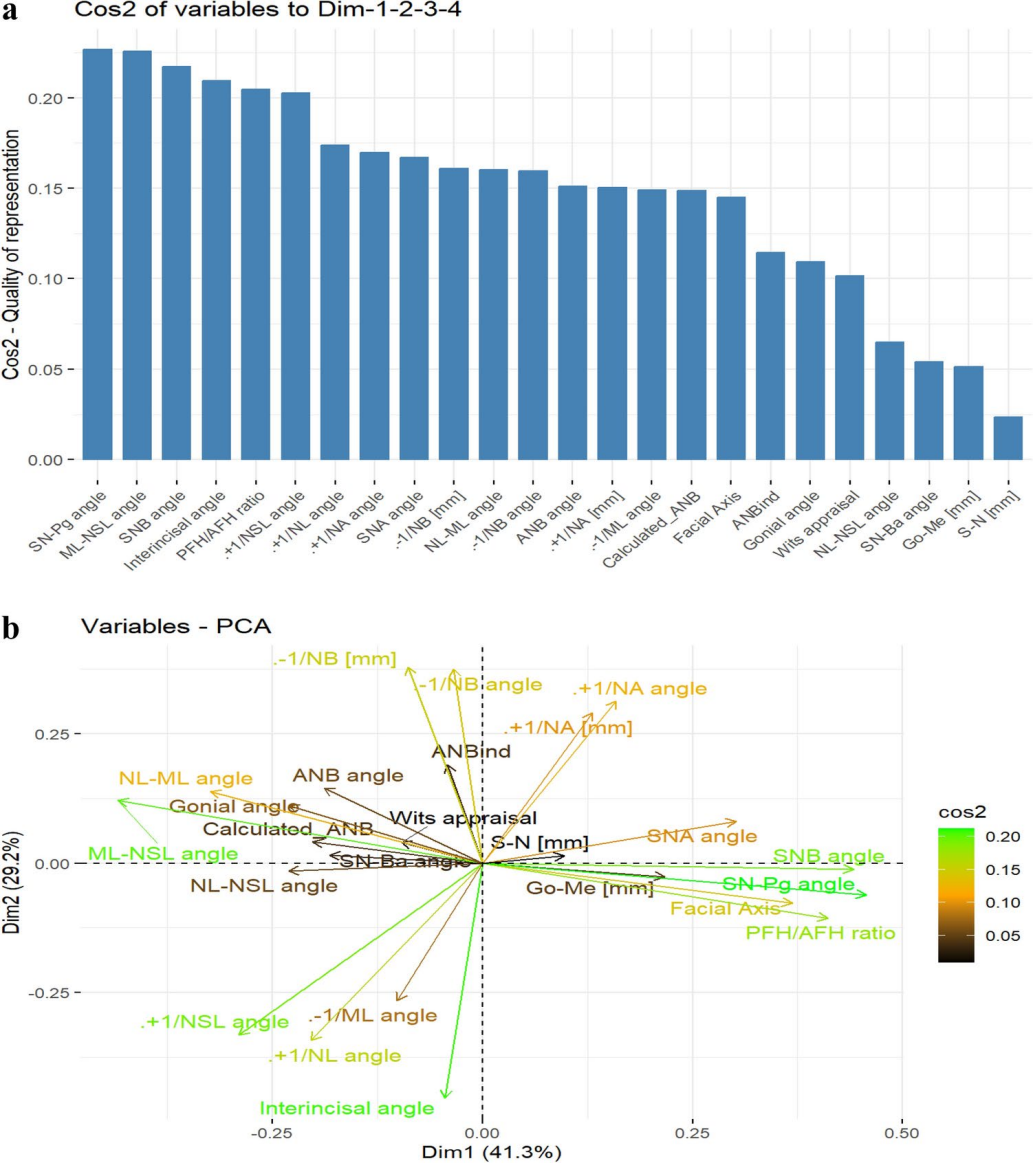
(**a**) Results of PCA showing the most relevant parameters in the diagnosis skeletal class I/ III. (**b**) Results of PCA showing the most relevant parameters in diagnosing skeletal class I/ III, including the direction of impact.

The PCA results are presented in Tables 3, 4 and Figs. 2a-b. According to the findings, the first principal component explains 41% of the variance in skeletal class I and III patients, whereas the first four principal components can explain 93% of the variance (Table 3).

**Table 3:** Variance in the diagnosis skeletal class I/ III explained by the first four Principal Components.

	Component 1	Component 2	Component 3	Component 4
Standard deviation	1.25	1.05	0.75	0.54
Proportion of variance	0.41	0.29	0.15	0.08
Cumulative proportion	0.41	0.70	0.85	0.93

**Table 4:** Loading of the cephalometric variables on the first four principal components.

Parameter	Component 1	Component 2	Component 3	Component 4
SNA angle	0.24	0.08	0.25	**0.34**
SNB angle	**0.35**	− 0.01	0.00	0.27
ANB angle	− 0.15	0.14	**0.40**	0.13
ANBind	− 0.03	0.18	0.11	**0.48**
Calculated_ANB	− 0.16	0.04	**0.41**	− 0.19
Wits appraisal	− 0.08	0.04	**0.39**	− 0.12
S-N [mm]	0.08	0.01	− 0.03	− 0.21
Go-Me [mm]	0.17	− 0.02	− 0.06	− 0.06
NL-ML angle	− 0.26	0.13	− 0.17	0.26
NL-NSL angle	− 0.18	− 0.01	− 0.03	− 0.19
ML-NSL angle	− **0.35**	0.12	− 0.18	0.13
PFH/AFH ratio	**0.33**	− 0.10	0.19	− 0.11
Gonial angle	− 0.18	0.11	− 0.22	0.23
Facial axis	0.29	− 0.07	0.00	− 0.10
SN-Ba angle	− 0.15	0.01	− 0.01	− 0.26
SN-Pg angle	**0.37**	− 0.06	− 0.01	0.22
+ 1/NL angle	− 0.16	− **0.33**	0.14	0.10
+ 1/NSL angle	− 0.23	− **0.32**	0.12	0.01
+ 1/NA angle	0.13	**0.30**	− 0.25	− 0.18
+ 1/NA [mm]	0.10	0.28	− 0.25	− 0.20
− 1/ML angle	− 0.08	− 0.25	− **0.31**	0.21
− 1/NB angle	− 0.03	**0.36**	0.17	0.03
− 1/NB [mm]	− 0.07	**0.36**	0.11	0.08
Interincisal angle	− 0.04	− 0.43	− 0.02	0.07

Among the cephalometric parameters, skeletal sagittal, skeletal vertical, and dental variables showed high loading values on the first four principal components. Concerning the first principal component, high positive loadings were observed for the growth pattern PFH/AFH (0.35), the mandible’s degree of prognathism SNB (0.35), the sagittal position of the chin SN-Pg (0.37), and a high negative loading was detected for the inclination of the mandible ML-NSL (− 0.35). The second principal component was highly affected by dental parameters (+ 1/NL, + 1/NSL, + 1/NA, − 1/NB), whereas the third and fourth principal components were predominantly influenced by skeletal sagittal and dental parameters (PC 3: ANB, Calculated ANB, Wits, − 1/ML; PC 4: SNA, and ANBind). The cosine square function (Fig. 2a) demonstrates that the four most relevant parameters in skeletal class I/ III explaining variance are the sagittal position of the chin (SN-Pg), mandible’s inclination (ML-NSL), SNB angle, interincisal angle, the growth pattern (PFH/AFH), and + 1/NSL angle with decreasing importance. As shown in Fig. 2b, the PCA biplot visualizes the relationships between the variables and the first two PCs (PC1 and PC2).

### Machine learning classification

**Fig. 3:**
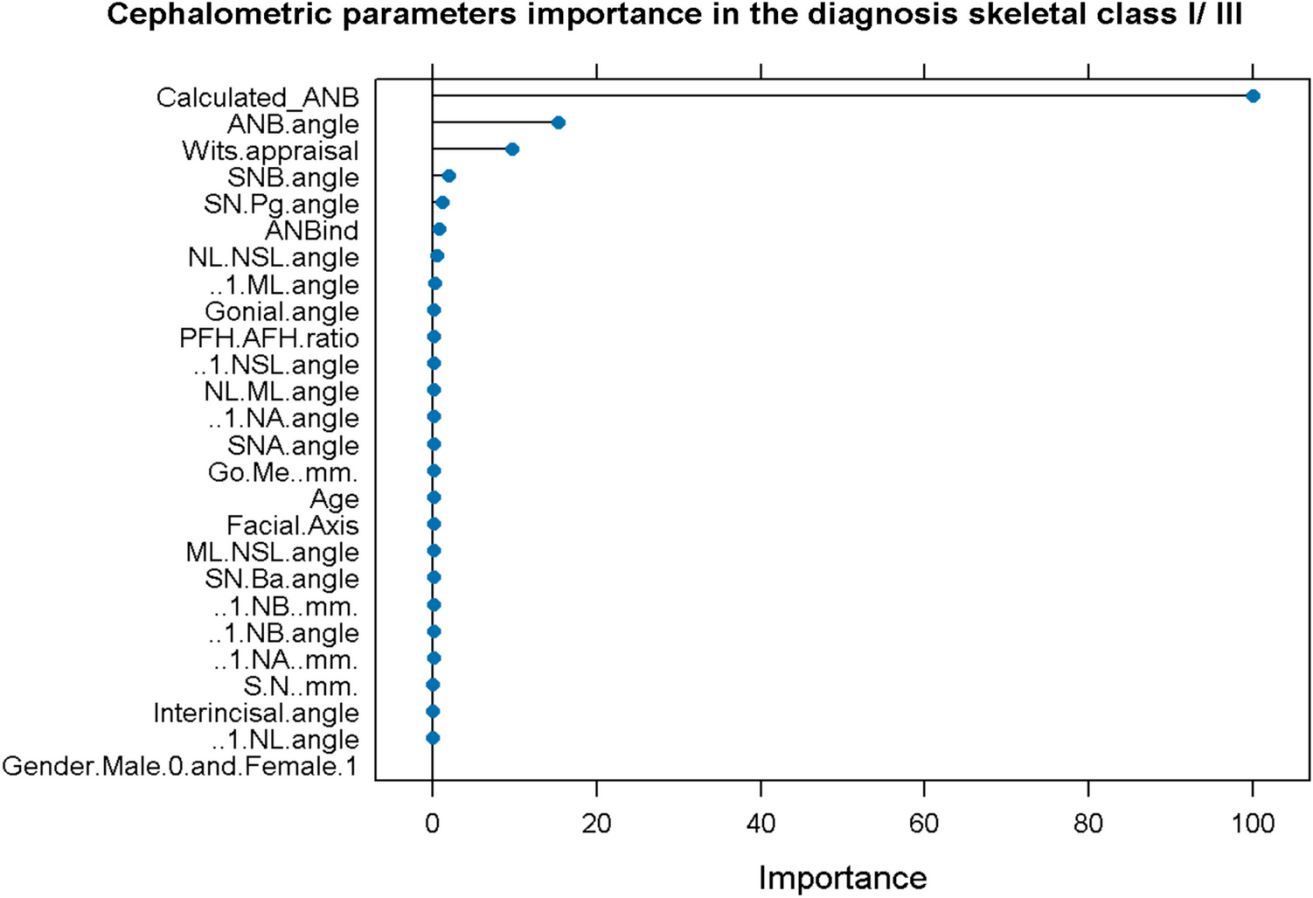
Importance of input variables in the diagnosis skeletal class I/III.

The general model, which included all cephalometric parameters and age and sex, was used to determine the importance of all input variables in determining skeletal class I/ III (Fig. 3). It can be seen that Wits appraisal, SNB, and SN-Pg were the most relevant variables when excluding ANB, ANBind, and Calculated_ANB.

Based on this information, various machine learning models with different input variables were evaluated. When neglecting Calculated_ANB and ANB angle, the following most relevant variable was Wits appraisal, which was the only input variable in model 1. Considering the following most important parameter, SNB angle, model 2 consisted of Wits appraisal and SNB angle only, whereas model 3 included also SN-Pg angle. Furthermore, a model was established, considering all parameters except for ANB, ANB individual, and Calculated_ANB.

The accuracy and reliability (kappa) of the different machine learning models obtained from the cross-validation data are reported in Table 5.

**Table 5:** This table presents the models’ performance (LDA, CART, KNN, SVM, RF, GLM) on the original data sample size and on the down-sampled balanced models.

	Wits	SNB	SN-Pg	Model (s)	Hyperparameters	Mean accuracy	Mean kappa	Balanced model (s)	Balanced models hyperparameters	Mean accuracy	Mean kappa
All parameters included-general model				LDA		0.90	0.77	LDA		0.87	0.74
				CART		1.00	1.00	CART		1.00	1.00
				KNN	KNN (k = 9)	0.87	0.70	KNN	KNN (k = 5)	0.87	0.74
				SVM	SVM (Kernel type = Radial Basis Function, Sigma = 0.027, C = 1)	0.92	0.81	SVM	SVM (Kernel type = Radial Basis Function, Sigma = 0.027, C = 1)	0.91	0.839
				RF		1.00	1.00	RF		1.00	1.00
				GLM		0.94	0.88	GLM		0.91	0.82
All parameters included except ANB, ANB individual and calculated_ANB				LDA		0.91	0.79	LDA		0.87	0.75
				CART		0.82	0.57	CART		0.83	0.67
				KNN	KNN (k = 7)	0.82	0.57	KNN	KNN (k = 5)	0.80	0.60
				SVM	SVM (Kernel type = Radial Basis Function, Sigma = 0.03, C = 1)	0.879	0.70	SVM	SVM (Kernel type = Radial Basis Function, Sigma = 0.03, C = 0.5)	0.86	0.73
				RF		0.85	0.63	RF		0.84	0.68
				GLM		0.94	0.88	GLM		0.91	0.83
Model 1	( +)	( −)	( −)	LDA		0.82	0.57	LDA		0.81	0.62
	( +)	( −)	( −)	CART		0.79	0.51	CART		0.83	0.67
	( +)	( −)	( −)	KNN	KNN (k = 9)	0.81	0.55	KNN	KNN (k = 9)	0.80	0.61
	( +)	( −)	( −)	SVM	SVM (Kernel type = Radial Basis Function, Sigma = 14.71, C = 0.25)	0.81	0.56	SVM	SVM (Kernel type = Radial Basis Function, Sigma = 11.75, C = 1)	0.81	0.63
	( +)	( −)	( −)	RF		0.80	0.54	RF		0.76	0.53
	( +)	( −)	( −)	GLM		0.83	0.60	GLM		0.82	0.64
Model 2	( +)	( +)	( −)	LDA		0.84	0.63	LDA		0.86	0.73
	( +)	( +)	( −)	CART		0.82	0.59	CART		0.84	0.68
	( +)	( +)	( −)	KNN	KNN (k = 5)	0.88	0.73	KNN	KNN (k = 9)	0.88	0.77
	( +)	( +)	( −)	SVM	SVM (Kernel type = Radial Basis Function, Sigma = 1.49, C = 0.5)	0.86	0.67	SVM	SVM (Kernel type = Radial Basis Function, Sigma = 1.28, C = 1)	0.88	0.77
	( +)	( +)	( −)	RF		0.84	0.64	RF		0.86	0.72
	( +)	( +)	( −)	GLM		0.85	0.67	GLM		0.88	0.76
Model 3	( +)	( +)	( +)	LDA		0.86	0.65	LDA		0.86	0.73
	( +)	( +)	( +)	CART		0.82	0.57	CART		0.83	0.669
	( +)	( +)	( +)	KNN	KNN (k = 7)	0.868	0.69	KNN	KNN (k = 7)	0.86	0.73
	( +)	( +)	( +)	SVM	SVM (Kernel type = Radial Basis Function, Sigma = 0.78, C = 1)	0.86	0.67	SVM	SVM (Kernel type = Radial Basis Function, Sigma = 1.07, C = 0.25)	0.85	0.72
	( +)	( +)	( +)	RF		0.85	0.65	RF		0.83	0.669
	( +)	( +)	( +)	GLM		0.87	0.70	GLM		0.85	0.71

The results in this table are obtained from the cross-validation data. For each model, the table included the predictors, important hyperparameters, the accuracy, and the reliability (kappa) results in diagnosing skeletal class I/ III.

The general model, which included all parameters, achieved perfect accuracy and reliability (kappa) for diagnosing skeletal class I/ III (1.00) with the model’s RF and CART. Considering only the Wits appraisal as an input variable (model 1) leads to a mean accuracy of 0.83 and a mean kappa of 0.60 with the GLM model. The performance was improved by taking Wits appraisal and SNB into account: here, accuracy and kappa increased to 0.88 and 0.73, respectively, when applying the KNN model. However, adding a further parameter, SN-Pg did not improve accuracy and kappa, which were equal to 0.87 and 0.70, respectively, in the GLM model.

**Fig. 4:**
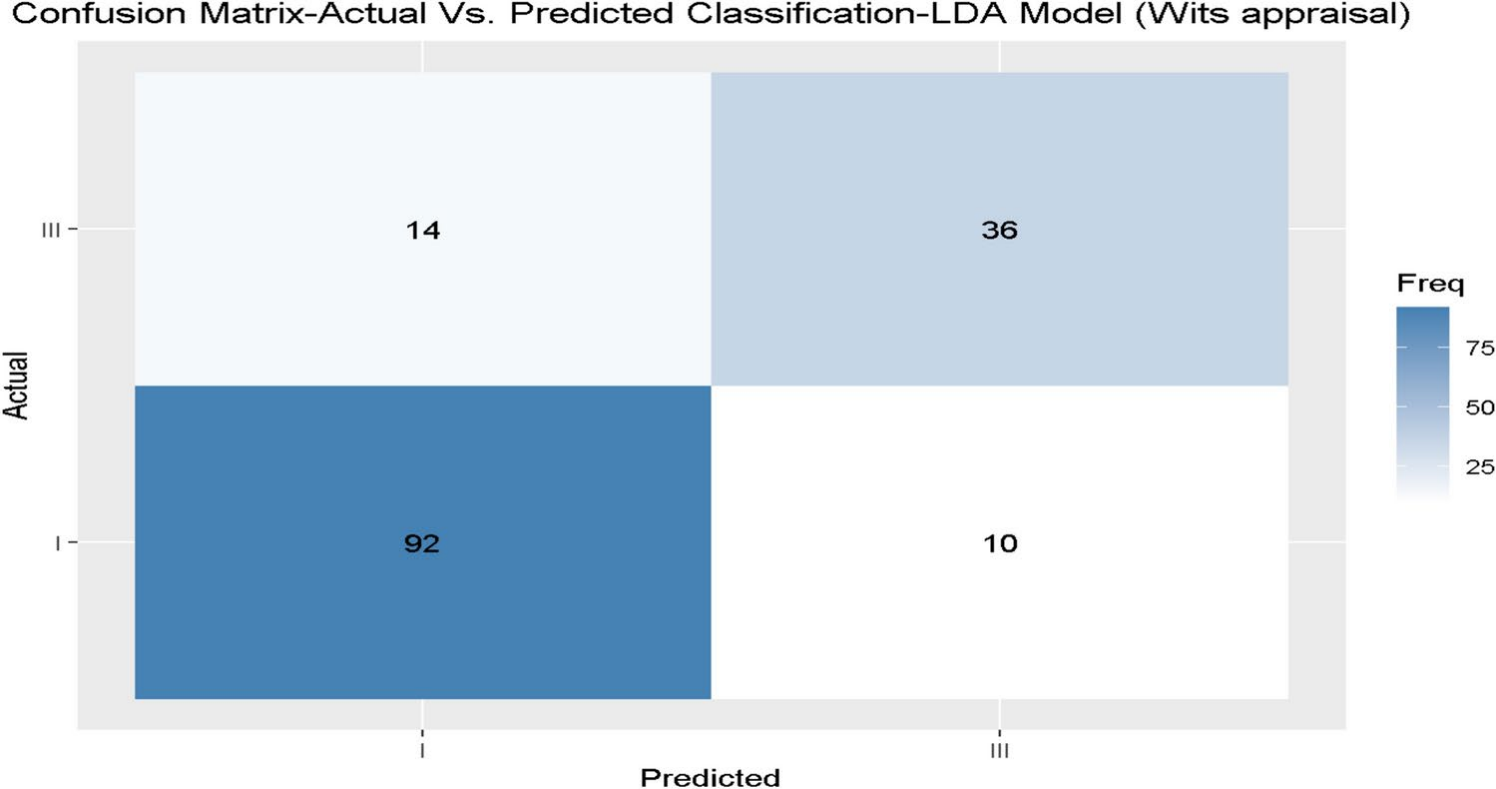
Independent validation data of model 1 (Wits only) for the diagnosis skeletal class I/III.

**Fig. 5:**
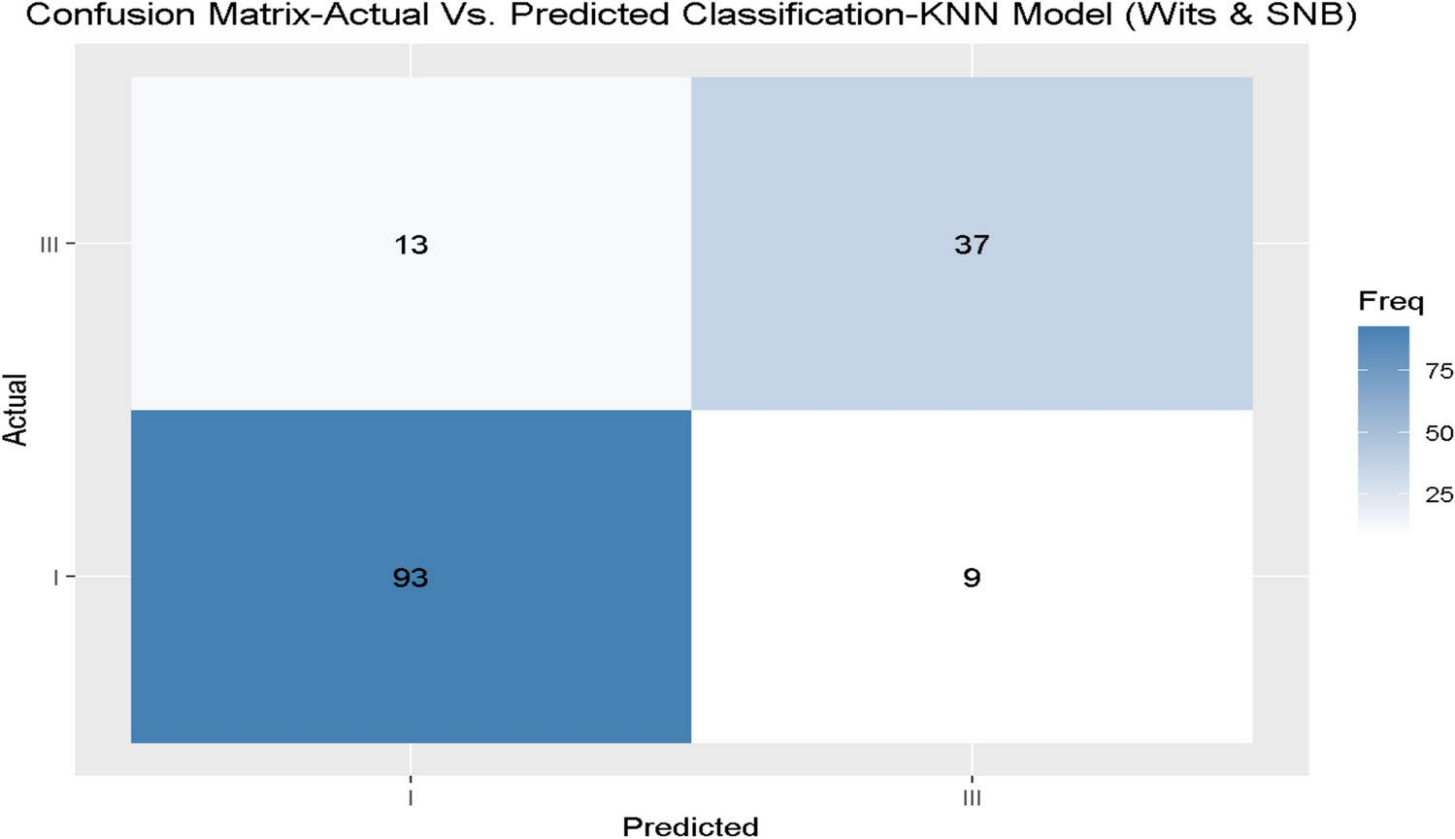
Independent validation data of model 2 (Wits and SNB only) for the diagnosis skeletal class I/III.

During the validation of the best fitting models, we presented the confusion matrix models 1 and 2, using the unseen independent validation set, as presented in Figs. 4 and 5 for models 1 and 2, respectively.

Finally, we repeated the analysis using balanced models and found that in the general model, the results showed the same accuracy (i.e., perfect accuracy and reliability (kappa)). However, when comparing the balanced model that included wits appraisal only, the GLM and LDA accuracy decreased slightly to 0.82 and 0.81, respectively. On the other hand, the CART model accuracy improved to 0.83. Regarding balanced model 2, the results showed an improvement in the LDA, CART, SVM, and GLM models. Finally, in model 3, the results showed improvement only on the CART model, as shown in Table 5.

### Machine learning classification (using the ANB angle and ANB_ind_^7^ input variables)

**Fig. 6:**
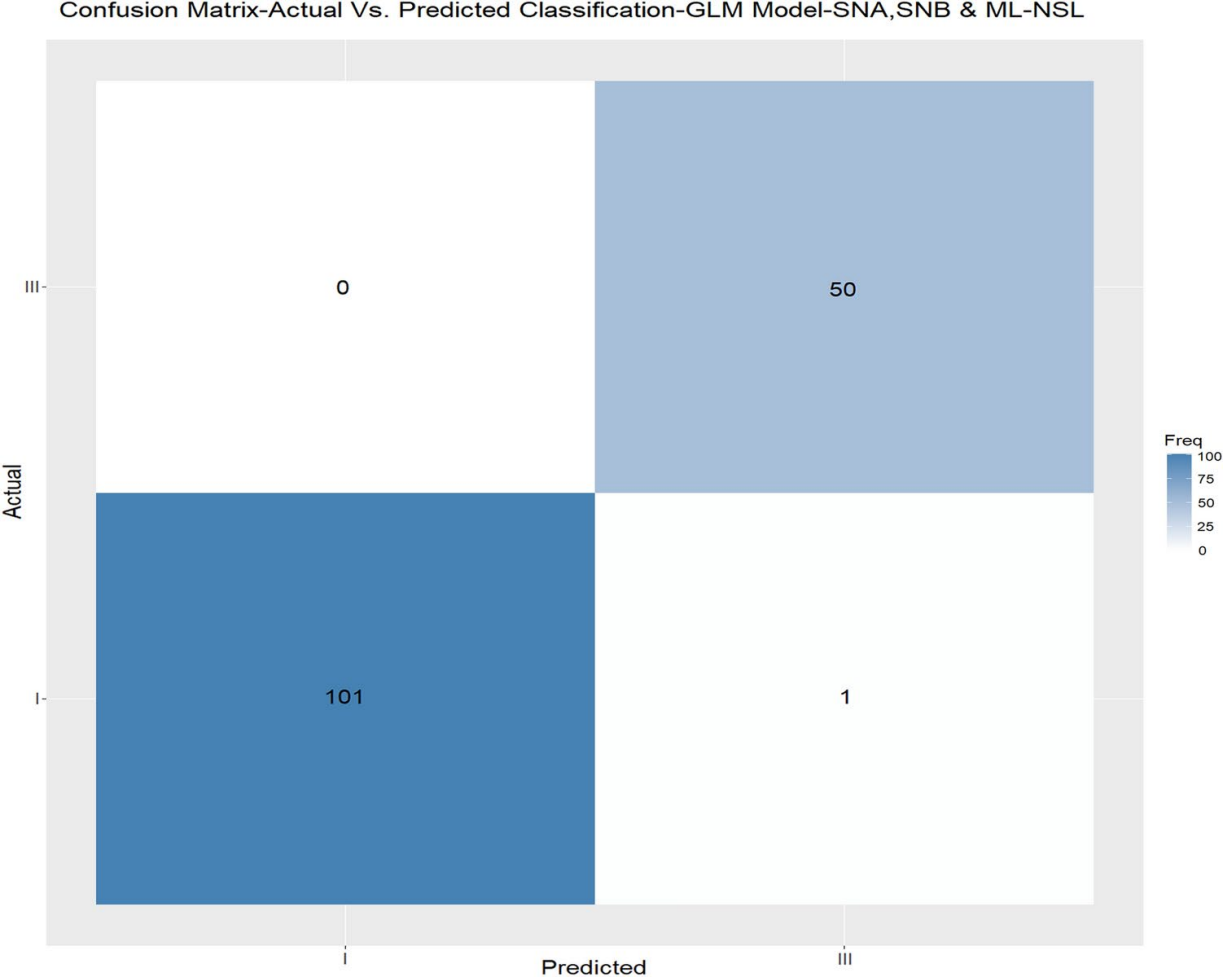
Independent validation data of the classification models based on SNA, SNB, and ML-NSL angles for the diagnosis of skeletal class I/III.

The machine learning models in the previous section demonstrated that the most crucial parameters in the classification process of skeletal class I and III were the parameters Calculated_ANB, ANB angle, and Wits appraisal. In addition, Wits appraisal only demonstrated the ability of machine learning models to classify skeletal class I/III malocclusion patients with a high accuracy of 0.83. In this section, we applied different machine learning classification models and examined the ability of the parameters that define ANB angle (i.e., SNA-SNB) and ANB_ind_ (i.e., the equation ANB_ind_ = -35.16 + 0.4 × SNA + 0.2 × ML-NSL^7^), the parameters- SNA, SNB, and ML-NSL angles to predict the skeletal classification as class I or III. The accuracy and reliability (kappa) of the different machine learning models that were obtained from the cross-validation data are reported in Table 6. The best model in this section was the GLM model, which gained approximately an accuracy of 0.99 (Accuracy = 0.988, Kappa = 0.97), followed by the LDA and SVM models (Accuracy = 0.92, Kappa = 0.81). Also, we presented the confusion matrix for the GLM model using the unseen independent validating data, as presented in Fig. 6.

**Table 6:** This table represents the classification models based on SNA, SNB, and ML-NSL angles for the diagnosis of skeletal class I/ III.

	SNA	SNB	ML-NSL	Hyperparameters	Mean accuracy	Mean kappa	Balanced models hyperparameters	Balanced model (s) mean accuracy	Balanced model (s) mean Kappa
LDA	(+)	( +)	( +)		0.92	0.81		0.95	0.91
CART	(+)	(+)	(+)		0.759	0.41		0.68	0.36
KNN	(+)	(+)	(+)	KNN (k = 9)	0.88	0.64	KNN (k = 7)	0.91	0.83
SVM	(+)	(+)	(+)	SVM (Kernel type = Radial Basis Function, Sigma = 0.65, C = 1)	0.92	0.81	SVM (Kernel type = Radial Basis Function, Sigma = 0.81, C = 1)	0.92	0.85
RF	(+)	(+)	(+)		0.88	0.75		0.85	0.71
GLM	(+)	(+)	(+)		0.988	0.97		0.99	0.98

For each model (LDA, CART, KNN, SVM, RF, GLM), the table summarized the accuracy and reliability (kappa) of six different machine learning models in diagnosing skeletal class I/III on the original sample size and on the down sampled data. The results in this table are obtained from the cross-validation data.

Finally, we repeated the analysis using balanced models here and received an improved GLM model with 0.99 accuracy, LDA with 0.95 accuracy, and KNN with 0.91 accuracy (Table 6).

## Discussion

Within this study, various reliable and valid machine learning models with differences in the number of input variables and the type of algorithm applied were established for diagnosing skeletal class I and III in German orthodontic patients by comparing the AI method with the gold standard, i.e., the manually determined Calculated_ANB. The statistical evaluation of the machine learning models included the analysis of the model’s accuracy, reliability (kappa), sensitivity, and specificity. As secondary outcomes, cephalometric measurements and their correlations were analyzed and compared between skeletal class I and III subjects and several age and sex-specific subgroups, revealing some significant differences. Furthermore, a PCA was conducted to identify the most important parameters in skeletal class I/III diagnosis.

### Cephalometric measurements

Analysing the effects of the confounding factors age and sex on cephalometric measurements, our results revealed that age caused more often differences than sex in both skeletal class I and III. In skeletal class I, adolescents presented a more horizontal growth pattern as shown by a bigger PFH/AFH ratio, smaller Gonial and NL-ML angles, and a higher degree of prognathism of the mandible and chin, which was evident from higher SNB and SN-Pg angles, than children. The forwardly directed growth of the mandible (SNB, SN-Pg) and the increase in PFH/AFH ratio were also described by other authors for patients of similar age with good occlusion^28–30^. However, the age limits used in the different investigations were not identical (Yoon and Chung: 9–18 years^29^, Bishara et al.: 5–10 years, 10–15 years, 15–25.5 years, 15–17 years, 17–25.5 years^28^), which does not allow a precise balancing of the findings^30^. Some authors found significant growth-related changes in SNB in males^30^ and others in females^28^, so the impact of sex on the mandible’s sagittal growth appears to depend on the population evaluated. Next, the higher proclination of upper incisors in males compared to females did not present statistical significance in all variables assessing this (+ 1/NL) and were relatively small (1.8–2°) and hence regarded as clinically irrelevant. In both skeletal classes investigated, our study did not reveal significant changes in SNA, i. e. the degree of maxillary prognathism, which is confirmed by other investigations based on skeletal class III^31^ and I^30^.

Patients with skeletal class III presented increased sagittal jaw discrepancy at higher ages, especially in males, as evident from a decrease in ANB angle and Calculated_ANB. Similarly, other investigations also observed a reduction in ANB angle from childhood towards (late) adolescence in class III subjects^31,32^. In our previous study, which compared Arab skeletal class II and III patients, Calculated_ANB was also smaller in older patients, i.e., skeletal class III severity was more pronounced^19^. In contrast to the class III results of this study, which presented significant differences for ANB and Calculated_ANB only, the previous investigation reported more age and sex-related differences in class III, e.g., a more horizontal growth pattern in males^19^. Such differences might be explained by the other population analysed (Arab vs. German). Contrary to an investigation performed by Baccetti et al.^33^, we did not observe only sex-related differences in cephalometric parameters in class III subjects because these were significant only for different age groups. A possible explanation might be that Baccetti et al.^33^ assessed measurements at partly smaller age ranges. Furthermore, we could not confirm the significant vertical growth pattern at higher ages in class III patients, which was described by Baccetti et al.^32^. However, Baccetti et al.^32^ investigated a smaller population (n = 22) with slightly different time points of assessment (on average 8 vs. 15 years).

Looking at skeletal class I and III, a more prognathic mandible (SNB) was observed in the latter group and this was reinforced if adults were part of the comparison. Similar findings have been reported in the literature: for example, Mitani et al. described a significantly more anterior chin position in class III than in class I^34^. Our results were partly contradictory concerning vertical measurements: whereas the facial axis indicated a more horizontal growth pattern, the Gonial angle implied a more vertical direction in skeletal class III children and adolescents. Aristide et al. also detected the higher Gonial angle found in class III in patients after the growth spurt^35^. Dental parameters demonstrated a dentoalveolar compensation of the skeletal sagittal dysgnathia in skeletal class III compared to class I: the upper incisors showed a bigger proclination and anterior position and the lower front teeth were more retroinclined. An investigation conducted on adolescent Saudis reported significantly more retroinlinced incisors in class III than in class I too, whereas the upper front teeth were significantly more proclined in females only^36^.

The correlation analysis revealed similar results for both skeletal classes investigated. The strong negative correlation between the growth pattern PFH/AFH and the mandible’s inclination ML/NSL describes that the growth pattern becomes more horizontal as the mandible is counterclockwise rotated. The positive associations found between the degree of prognathism of both jaws (SNB, SNA) describe that generally, the upper and lower jaw follow a similar pattern in the sagittal direction. The negative correlation between the mandible’s degree of prognathism (SNB) and inclination (ML/NSL) demonstrates also the topographical relation between these two parameters, which results in a more retrognathic lower jaw in case of its posterior rotation. In line with these observations, Segner described a positive correlation between SNA and SNB and a negative one between SNB and ML-NSL in patients with ideal occlusion^11^. Significant correlations were also observed for Calculated_ANB and several other parameters, showing that the diagnosis of the skeletal class is influenced by more parameters than the antero-posterior distance between points A and B. Negative correlations were found with SNB and SN-Pg. In contrast, positive associations were observed with mandibular length, which clarifies the impact of the mandible’s sagittal position on skeletal class I/ III. Additionally, it was positively associated with ANB and Wits appraisal in both classes but negatively with the Facial axis in class III and with the maxilla’s inclination (NL-NSL) in class I only. Similarly, the study on Arab class III patients showed a correlation between Calculated_ANB with Facial axis, SNB, SN-Pg, ANB, and Wits appraisal, despite the different populations^19^. The associations between the maxilla’s inclination and the facial axis were also described for the individualised ANB in another investigation^37^.

The PCA revealed that the four first principal components could explain 93% of the total variance in skeletal class I/ III diagnosis, which is comparable to the result obtained in Arab patients (92%)^19^. The first principal component, which explained 41% of the variance in class I/ III diagnosis, was mainly influenced by the growth pattern (PFH/AFH) and the vertical (ML-NSL) as well as the sagittal position (SNB, SN-Pg) of the mandible and associated variables. The same message can be made based on the cosine square function and biplot (Fig. 2) and demonstrates the high influence of the lower jaw on skeletal class I/ III diagnosis and the importance of the correct identification of the corresponding cephalometric landmarks.

With RF and CART, the general machine learning model resulted in perfect accuracy and reliability, showing its suitability to define an individual as skeletal class I or III automatically. This finding is not surprising due to the principle of these machine learning models, which is, in a simplified description, a replication of the equation for the individualised ANB of Panagiotidis and Witt. Still, the advantage of our method compared to the gold standard is the automated process of diagnosing skeletal class I/ III, which might be useful in automated diagnoses, which are increasingly developed. The general model revealed that Wits appraisal, SNB, and SN-Pg were the most important factors in skeletal class I/ III diagnosis, when excluding Calculated_ANB and ANB, which is identical to our previous investigation^19^. Comparing the performances of models 1 to 3 to reduce the amount of input variables, our results identified KNN-model 2 (Accuracy = 0.88, Kappa = 0.73) to be better than model 1 (Accuracy = 0.83, Kappa = 0.60) and model 3 (Accuracy = 0.87, Kappa = 0.70), when considering both accuracy and reliability. In the last section of the machine learning classification, we applied different machine learning classification models, and examined the ability of the parameters that define ANB angle (i.e., SNA-SNB) and ANB_ind_ (i.e., the equation ANB_ind_ = -35.16 + 0.4 × SNA + 0.2 × ML-NSL^7^), the parameters- SNA, SNB, and ML-NSL angles to predict the classification as skeletal class I or III, and the results demonstrated that the GLM model gained approximately accuracy of 0.99 (Accuracy = 0.988, Kappa = 0.97), followed by the LDA and SVM models (Accuracy = 0.92, Kappa = 0.81). This performance was better than the gold standard individualized ANB of Panagiotidis and Witt, which includes three parameters (ANB, SNA, ML-NSL), and also better than our recently published article which was achieved by model 2 (SVM) for skeletal class II/ III diagnosis of Arab patients (Accuracy = 0.95, Kappa = 0.91)^19^. In another study that was published recently by Midlej et al.^38^ and examined the ability of machine learning applications to diagnose Arab patients as skeletal class I or II, found that the general machine learning model that included all measurements for patient classification showed a classification accuracy of 0.87 in the Random Forest and the Classification and Regression Tree models. In addition, the same study found that by using ANB angle and Wits appraisal only, an accuracy of 0.78 was achieved to classify patients as skeletal class I or II. One more study was done by Zhou et al., which examined 408 X-ray lateral cephalograms from Chinese patients. After image processing and feature engineering, nine supervised machine learning algorithms were applied for sagittal and vertical skeletal patterns. The multi-layer perceptron model was the most accurate model, which achieved 97.56% accuracy for sagittal pattern^39^ in the study performed by Niño-Sandoval et al. in 229 lateral cephalograms from Colombian young. The results demonstrated an accuracy of 74.51% in the Support Vector Machine with a linear kernel classifier^40^ in determining patients’ skeletal class compared to the gold standard ANB angle. The differences in accuracy between the reported studies and our results could be explained by variations concerning the population (age, ethnicity), the machine learning model and input variables used, and the reference standard applied.

Establishing the machine learning model for skeletal class I/ III diagnosis might be helpful and time-saving in clinical practice when combining it with an automated identification of cephalometric landmarks, which achieves reliable results^41^. Furthermore, it can be regarded as a feasibility study, showing that the machine learning model is a promising method that needs further development in larger populations.

## Limitations

This investigation suffers from some limitations. First, most of the patients recruited were between 0 and 13 years old, followed by adolescents. In contrast, adults presented only a minority of the study pool (n = 14 in skeletal class I, n = 18 in skeletal class III). This inhomogeneous age distribution can be explained by the retrospective stratification of patients collected within the recruitment time period. However, this disadvantage needs to be considered during interpretation, especially of age and sex-specific subgroups and must be optimized in future studies. In addition, this study enrolled 341 Class I vs. 168 Class III subjects, which means that some of the analysis can be biased due to moderately imbalanced groups. To deal with this limitation, we performed the down sampling analysis in the machine learning models to minimize the effect of the imbalanced groups. Furthermore, this analysis considered only skeletal class I and III but not class II and might be leaded to overfitting in the machine learning models. This concern is approached by upcoming studies, which will extend the study population.

## Conclusion and future research

Within this investigation, we established a new machine learning model that can successfully diagnose German orthodontic patients as skeletal class I and III. The parameters- SNA, SNB, and ML-NSL angles were able to predict the classification with almost perfect accuracy, and the results demonstrated that the GLM model gained approximately accuracy of 0.99 (Accuracy = 0.988, Kappa = 0.97), followed by the LDA and SVM models (Accuracy = 0.92, Kappa = 0.81). The model can be applied within feasibility studies requiring significantly fewer input variables and in daily routine in combination with automated identification of the corresponding reference landmarks. In the bottom line, these results are one more step towards the incorporation of artificial intelligence techniques in the diagnosis and treatment process among orthodontic patients and will enable orthodontists to classify patients accurately by incorporating artificial intelligence models along with the clinical diagnosis process and thus can prevent misclassifications and inaccurate treatment plans. A review by Bichu et al. 42 about applications of artificial intelligence and machine learning in orthodontics found that the most commonly studied domains in this field were diagnosis and treatment planning. Furthermore, the PCA revealed the high importance of some variables in explaining skeletal class I/III variance. These are the sagittal position of the chin (SN-Pg), mandible’s inclination (ML-NSL), SNB angle, interincisal angle, the growth pattern (PFH/AFH), and + 1/NSL angle. However, future studies analyzing a broader population are necessary to investigate the effects of age and sex on cephalometric measurements and their correlations in more detail and to test the performance of the machine learning model presented. Furthermore, future research should investigate all skeletal classes and apply different machine learning and deep-learning models. Finally, further research should include a larger sample size from various ethnic populations, that will help validate these models worldwide.

## Supplementary Information

Supplementary Information.

## Data Availability

The datasets used and/or analysed during the current study available from the corresponding author on reasonable request.
