# Supplementary material for: Machine learning models for improving the diagnosing efficiency of skeletal class I and III in German orthodontic patients
Source: Sci Rep. 2025 Apr 13;15:12738. doi: 10.1038/s41598-025-97717-6 (PMC11994767; doi:10.1038/s41598-025-97717-6)
Supplement: Supplementary file 1 — Supplementary Information. [file 41598_2025_97717_MOESM1_ESM.pdf]

Supplementary Table 1.

	variable	definition
skeletal sagittal	SNA [°]	caudal angle between Sella, Nasion and point A
	SNB [°]	caudal angle between Sella, Nasion and point B
	ANB [°]	caudal angle between Nasion, point A and point B
	NSBa [°]	anterior angle between Nasion, Sella and Basion
	SNPg [°]	caudal angle between Sella, Nasion and Pogonion
	SN [mm]	linear distance between the points Sella and Nasion, describing the anterior skull base
	GoMe [mm]	linear distance between the points Gonion and Menton, describing the length of the mandible
	Wits [mm]	linear distance between perpendiculars from point A and point B on the occlusal plane, describing skeletal class
skeletal vertical	NL-ML [°]	anterior angle between the lines NL (Spa-Spp) and ML (T2-Me), describing the divergence of the jaw bases
	ML-NSL [°]	anterior angle between the lines NSL (Nasion-Sella) and ML, describing the inclination of the mandible
	NL-NSL [°]	anterior angle between lines NL and NSL, describing the inclination of the maxilla
	PFH/AFH [%]	ratio between the posterior (PFH = distance Sella-Gonion) and anterior (AFH = distance Nasion-Menton) facial height
	Gonial angle [°]	anterior angle between the lines ML and GoAr
	Facial axis [°]	caudal angle between the lines NBa and PtGN’
dental	+1/NL [°]	anterior angle between the upper incisor’s tooth axis and the line NL
	+1/NSL [°]	anterior angle between the upper incisor’s tooth axis and the line NSL
	+1/NA [°]	caudal angle between the upper incisor’s tooth axis and the line NA
	+1/NA [mm]	linear perpendicular distance between the incisal edge of the upper incisor to line NA, describing its sagittal position
	-1/ML [°]	anterior angle between the lower incisor’s tooth axis and the line ML, describing its inclination
	-1/NB [°]	caudal angle between the lower incisor’s tooth axis and the line NB, describing its inclination
	-1/NB [mm]	linear perpendicular distance between the lower incisor’s tooth axis and the line NB, describing its sagittal position
	Interincisal angle [°]	horizontal angle between the tooth axes of the upper and lower incisors, describing the relation of their inclination to each other

Supplementary Table 1. The main cephalometric parameters that were used in this study, including their visualisation in **Supplementary** Figure 1.

Supplementary Table 2.

		Shapiro Wilk Test			
Subgroup	Parameter	Statistic	Group size	Sig.	Normality
I_Female_Age>21	SNA [°]	0.95	8	0.716	Normal Distribution
I_Female_Age>21	SNB [°]	0.85	8	0.096	Normal Distribution
I_Female_Age>21	ANB [°]	0.949	8	0.696	Normal Distribution
I_Female_Age>21	ANB_{ind} [°]	0.943	8	0.636	Normal Distribution
I_Female_Age>21	Calculated_ANB [°]	0.841	8	0.077	Normal Distribution
I_Female_Age>21	Wits appraisal [mm]	0.898	8	0.277	Normal Distribution
I_Female_Age>21	S-N [mm]	0.741	8	0.007	Not Normal
I_Female_Age>21	Go-Me [mm]	0.923	8	0.458	Normal Distribution
I_Female_Age>21	NL-ML [°]	0.941	8	0.622	Normal Distribution
I_Female_Age>21	NL-NSL [°]	0.845	8	0.085	Normal Distribution
I_Female_Age>21	ML-NSL [°]	0.947	8	0.677	Normal Distribution
I_Female_Age>21	PFH/AFH [%]	0.981	8	0.969	Normal Distribution
I_Female_Age>21	Gonial_angle [°]	0.871	8	0.153	Normal Distribution
I_Female_Age>21	Facial axis [°]	0.883	8	0.202	Normal Distribution
I_Female_Age>21	NS-Ba [°]	0.772	8	0.014	Not Normal
I_Female_Age>21	SN-Pg [°]	0.919	8	0.419	Normal Distribution
I_Female_Age>21	+1/NL [°]	0.933	8	0.543	Normal Distribution
I_Female_Age>21	+1/NSL [°]	0.97	8	0.897	Normal Distribution
I_Female_Age>21	+1/NA [°]	0.967	8	0.871	Normal Distribution
I_Female_Age>21	+1/NA [mm]	0.939	8	0.602	Normal Distribution
I_Female_Age>21	-1/ML[°]	0.916	8	0.397	Normal Distribution
I_Female_Age>21	-1/NB [°]	0.86	8	0.12	Normal Distribution
I_Female_Age>21	-1/NB [mm]	0.879	8	0.184	Normal Distribution
I_Female_Age>21	Interincisal angle [°]	0.955	8	0.757	Normal Distribution
I_Male_14<Age<20	SNA [°]	0.962	25	0.448	Normal Distribution
I_Male_14<Age<20	SNB [°]	0.979	25	0.858	Normal Distribution
I_Male_14<Age<20	ANB [°]	0.921	25	0.055	Normal Distribution
I_Male_14<Age<20	ANB_{ind} [°]	0.926	25	0.071	Normal Distribution
I_Male_14<Age<20	Calculated_ANB [°]	0.92	25	0.052	Normal Distribution
I_Male_14<Age<20	Wits appraisal [mm]	0.971	25	0.68	Normal Distribution
I_Male_14<Age<20	S-N [mm]	0.96	25	0.412	Normal Distribution
I_Male_14<Age<20	Go-Me [mm]	0.977	25	0.816	Normal Distribution
I_Male_14<Age<20	NL-ML [°]	0.977	25	0.817	Normal Distribution
I_Male_14<Age<20	NL-NSL [°]	0.968	25	0.607	Normal Distribution
I_Male_14<Age<20	ML-NSL [°]	0.866	25	0.004	Not Normal
I_Male_14<Age<20	PFH/AFH [%]	0.904	25	0.022	Not Normal
I_Male_14<Age<20	Gonial_angle [°]	0.94	25	0.145	Normal Distribution
I_Male_14<Age<20	Facial axis [°]	0.961	25	0.439	Normal Distribution
I_Male_14<Age<20	NS-Ba [°]	0.988	25	0.988	Normal Distribution
I_Male_14<Age<20	SN-Pg [°]	0.957	25	0.36	Normal Distribution
I_Male_14<Age<20	+1/NL [°]	0.959	25	0.393	Normal Distribution
I_Male_14<Age<20	+1/NSL [°]	0.959	25	0.392	Normal Distribution
I_Male_14<Age<20	+1/NA [°]	0.984	25	0.956	Normal Distribution
I_Male_14<Age<20	+1/NA [mm]	0.964	25	0.494	Normal Distribution
I_Male_14<Age<20	-1/ML[°]	0.986	25	0.973	Normal Distribution
I_Male_14<Age<20	-1/NB [°]	0.978	25	0.837	Normal Distribution
I_Male_14<Age<20	-1/NB [mm]	0.97	25	0.645	Normal Distribution
I_Male_14<Age<20	Interincisal angle [°]	0.983	25	0.93	Normal Distribution
I_Male_Age>21	SNA [°]	0.883	6	0.284	Normal Distribution
I_Male_Age>21	SNB [°]	0.888	6	0.31	Normal Distribution
I_Male_Age>21	ANB [°]	0.944	6	0.69	Normal Distribution
I_Male_Age>21	ANB_{ind} [°]	0.958	6	0.804	Normal Distribution
I_Male_Age>21	Calculated_ANB [°]	0.933	6	0.602	Normal Distribution
I_Male_Age>21	Wits appraisal [mm]	0.989	6	0.986	Normal Distribution
I_Male_Age>21	S-N [mm]	0.977	6	0.935	Normal Distribution
I_Male_Age>21	Go-Me [mm]	0.939	6	0.652	Normal Distribution
I_Male_Age>21	NL-ML [°]	0.936	6	0.625	Normal Distribution
I_Male_Age>21	NL-NSL [°]	0.935	6	0.623	Normal Distribution
I_Male_Age>21	ML-NSL [°]	0.905	6	0.404	Normal Distribution

I_Male_Age>21	PFH/AFH [%]	0.964	6	0.849	Normal Distribution
I_Male_Age>21	Gonial_angle [°]	0.98	6	0.954	Normal Distribution
I_Male_Age>21	Facial axis [°]	0.928	6	0.562	Normal Distribution
I_Male_Age>21	NS-Ba [°]	0.974	6	0.92	Normal Distribution
I_Male_Age>21	SN-Pg [°]	0.896	6	0.348	Normal Distribution
I_Male_Age>21	+1/NL [°]	0.86	6	0.188	Normal Distribution
I_Male_Age>21	+1/NSL [°]	0.936	6	0.623	Normal Distribution
I_Male_Age>21	+1/NA [°]	0.9	6	0.375	Normal Distribution
I_Male_Age>21	+1/NA [mm]	0.954	6	0.775	Normal Distribution
I_Male_Age>21	-1/ML[°]	0.899	6	0.369	Normal Distribution
I_Male_Age>21	-1/NB [°]	0.915	6	0.471	Normal Distribution
I_Male_Age>21	-1/NB [mm]	0.866	6	0.21	Normal Distribution
I_Male_Age>21	Interincisal angle [°]	0.904	6	0.399	Normal Distribution
III_Female_14<Age<20	SNA [°]	0.83	13	0.016	Not Normal
III_Female_14<Age<20	SNB [°]	0.944	13	0.504	Normal Distribution
III_Female_14<Age<20	ANB [°]	0.94	13	0.459	Normal Distribution
III_Female_14<Age<20	ANB_{ind} [°]	0.98	13	0.977	Normal Distribution
III_Female_14<Age<20	Calculated_ANB [°]	0.894	13	0.111	Normal Distribution
III_Female_14<Age<20	Wits appraisal [mm]	0.983	13	0.991	Normal Distribution
III_Female_14<Age<20	S-N [mm]	0.909	13	0.18	Normal Distribution
III_Female_14<Age<20	Go-Me [mm]	0.787	13	0.005	Not Normal
III_Female_14<Age<20	NL-ML [°]	0.968	13	0.869	Normal Distribution
III_Female_14<Age<20	NL-NSL [°]	0.98	13	0.979	Normal Distribution
III_Female_14<Age<20	ML-NSL [°]	0.918	13	0.238	Normal Distribution
III_Female_14<Age<20	PFH/AFH [%]	0.922	13	0.263	Normal Distribution
III_Female_14<Age<20	Gonial_angle [°]	0.935	13	0.401	Normal Distribution
III_Female_14<Age<20	Facial axis [°]	0.862	13	0.041	Not Normal
III_Female_14<Age<20	NS-Ba [°]	0.957	13	0.708	Normal Distribution
III_Female_14<Age<20	SN-Pg [°]	0.942	13	0.482	Normal Distribution
III_Female_14<Age<20	+1/NL [°]	0.906	13	0.163	Normal Distribution
III_Female_14<Age<20	+1/NSL [°]	0.924	13	0.282	Normal Distribution
III_Female_14<Age<20	+1/NA [°]	0.915	13	0.215	Normal Distribution
III_Female_14<Age<20	+1/NA [mm]	0.951	13	0.613	Normal Distribution
III_Female_14<Age<20	-1/ML[°]	0.848	13	0.027	Not Normal
III_Female_14<Age<20	-1/NB [°]	0.967	13	0.86	Normal Distribution
III_Female_14<Age<20	-1/NB [mm]	0.959	13	0.734	Normal Distribution
III_Female_14<Age<20	Interincisal angle [°]	0.933	13	0.37	Normal Distribution
III_Female_Age>21	SNA [°]	0.901	10	0.223	Normal Distribution
III_Female_Age>21	SNB [°]	0.968	10	0.867	Normal Distribution
III_Female_Age>21	ANB [°]	0.901	10	0.226	Normal Distribution
III_Female_Age>21	ANB_{ind} [°]	0.859	10	0.074	Normal Distribution
III_Female_Age>21	Calculated_ANB [°]	0.766	10	0.006	Not Normal
III_Female_Age>21	Wits appraisal [mm]	0.903	10	0.234	Normal Distribution
III_Female_Age>21	S-N [mm]	0.971	10	0.897	Normal Distribution
III_Female_Age>21	Go-Me [mm]	0.852	10	0.061	Normal Distribution
III_Female_Age>21	NL-ML [°]	0.887	10	0.157	Normal Distribution
III_Female_Age>21	NL-NSL [°]	0.839	10	0.042	Not Normal
III_Female_Age>21	ML-NSL [°]	0.815	10	0.022	Not Normal
III_Female_Age>21	PFH/AFH [%]	0.861	10	0.077	Normal Distribution
III_Female_Age>21	Gonial_angle [°]	0.899	10	0.214	Normal Distribution
III_Female_Age>21	Facial axis [°]	0.969	10	0.878	Normal Distribution
III_Female_Age>21	NS-Ba [°]	0.932	10	0.465	Normal Distribution
III_Female_Age>21	SN-Pg [°]	0.897	10	0.205	Normal Distribution
III_Female_Age>21	+1/NL [°]	0.925	10	0.4	Normal Distribution
III_Female_Age>21	+1/NSL [°]	0.874	10	0.11	Normal Distribution
III_Female_Age>21	+1/NA [°]	0.946	10	0.622	Normal Distribution
III_Female_Age>21	+1/NA [mm]	0.951	10	0.675	Normal Distribution
III_Female_Age>21	-1/ML[°]	0.869	10	0.098	Normal Distribution
III_Female_Age>21	-1/NB [°]	0.903	10	0.234	Normal Distribution
III_Female_Age>21	-1/NB [mm]	0.917	10	0.336	Normal Distribution
III_Female_Age>21	Interincisal angle [°]	0.83	10	0.033	Not Normal
III_Male_14<Age<20	SNA [°]	0.964	18	0.676	Normal Distribution
III_Male_14<Age<20	SNB [°]	0.966	18	0.724	Normal Distribution

III_Male_14<Age<20	ANB [°]	0.945	18	0.351	Normal Distribution
III_Male_14<Age<20	ANB_{ind} [°]	0.965	18	0.695	Normal Distribution
III_Male_14<Age<20	Calculated_ANB [°]	0.827	18	0.004	Not Normal
III_Male_14<Age<20	Wits appraisal [mm]	0.951	18	0.438	Normal Distribution
III_Male_14<Age<20	S-N [mm]	0.948	18	0.396	Normal Distribution
III_Male_14<Age<20	Go-Me [mm]	0.983	18	0.974	Normal Distribution
III_Male_14<Age<20	NL-ML [°]	0.895	18	0.048	Not Normal
III_Male_14<Age<20	NL-NSL [°]	0.917	18	0.116	Normal Distribution
III_Male_14<Age<20	ML-NSL [°]	0.935	18	0.237	Normal Distribution
III_Male_14<Age<20	PFH/AFH [%]	0.913	18	0.096	Normal Distribution
III_Male_14<Age<20	Gonial_angle [°]	0.953	18	0.466	Normal Distribution
III_Male_14<Age<20	Facial axis [°]	0.97	18	0.805	Normal Distribution
III_Male_14<Age<20	NS-Ba [°]	0.939	18	0.279	Normal Distribution
III_Male_14<Age<20	SN-Pg [°]	0.968	18	0.759	Normal Distribution
III_Male_14<Age<20	+1/NL [°]	0.91	18	0.085	Normal Distribution
III_Male_14<Age<20	+1/NSL [°]	0.93	18	0.195	Normal Distribution
III_Male_14<Age<20	+1/NA [°]	0.974	18	0.87	Normal Distribution
III_Male_14<Age<20	+1/NA [mm]	0.909	18	0.084	Normal Distribution
III_Male_14<Age<20	-1/ML[°]	0.972	18	0.836	Normal Distribution
III_Male_14<Age<20	-1/NB [°]	0.96	18	0.604	Normal Distribution
III_Male_14<Age<20	-1/NB [mm]	0.898	18	0.053	Normal Distribution
III_Male_14<Age<20	Interincisal angle [°]	0.93	18	0.193	Normal Distribution
III_Male_Age>21	SNA [°]	0.986	8	0.987	Normal Distribution
III_Male_Age>21	SNB [°]	0.968	8	0.879	Normal Distribution
III_Male_Age>21	ANB [°]	0.896	8	0.265	Normal Distribution
III_Male_Age>21	ANB_{ind} [°]	0.972	8	0.913	Normal Distribution
III_Male_Age>21	Calculated_ANB [°]	0.902	8	0.303	Normal Distribution
III_Male_Age>21	Wits appraisal [mm]	0.965	8	0.855	Normal Distribution
III_Male_Age>21	S-N [mm]	0.874	8	0.163	Normal Distribution
III_Male_Age>21	Go-Me [mm]	0.929	8	0.508	Normal Distribution
III_Male_Age>21	NL-ML [°]	0.899	8	0.282	Normal Distribution
III_Male_Age>21	NL-NSL [°]	0.966	8	0.863	Normal Distribution
III_Male_Age>21	ML-NSL [°]	0.792	8	0.024	Not Normal
III_Male_Age>21	PFH/AFH [%]	0.804	8	0.032	Not Normal
III_Male_Age>21	Gonial_angle [°]	0.881	8	0.193	Normal Distribution
III_Male_Age>21	Facial axis [°]	0.966	8	0.862	Normal Distribution
III_Male_Age>21	NS-Ba [°]	0.915	8	0.393	Normal Distribution
III_Male_Age>21	SN-Pg [°]	0.974	8	0.929	Normal Distribution
III_Male_Age>21	+1/NL [°]	0.918	8	0.412	Normal Distribution
III_Male_Age>21	+1/NSL [°]	0.873	8	0.162	Normal Distribution
III_Male_Age>21	+1/NA [°]	0.944	8	0.65	Normal Distribution
III_Male_Age>21	+1/NA [mm]	0.969	8	0.894	Normal Distribution
III_Male_Age>21	-1/ML[°]	0.841	8	0.078	Normal Distribution
III_Male_Age>21	-1/NB [°]	0.898	8	0.276	Normal Distribution
III_Male_Age>21	-1/NB [mm]	0.914	8	0.382	Normal Distribution
III_Male_Age>21	Interincisal angle [°]	0.974	8	0.929	Normal Distribution

Supplementary Table 2. This table represents for every subgroup that is smaller than 30 individuals, the Shapiro-Wilk exam to test the normality. The table includes the subgroup, cephalometric parameter (parameter), Shapiro Wilk statistic (statistic), number of individuals within the subgroup (group size), Shapiro Wilk significance (sig), and normality result (normality).

Supplementary Table 3.

Variable	Mean		SD		Min		Pctl. 25		Pctl. 75		Max	
	I	III	I	III	I	III	I	III	I	III	I	III
SNA [°]	81	81	3.8	3.9	63	70	79	79	84	84	92	89
SNB [°]	78	81	3.2	3.7	66	71	76	78	80	83	87	92
ANB [°]	3.7	0.32	1.6	2.3	-2.5	-10	2.6	-0.8	4.7	1.9	9	4.9
ANB_{ind} [°]	3.6	3.6	1.4	1.5	-3.2	-0.72	2.8	2.7	4.5	4.7	7.8	7.2
Calculated_ANB [°]	0.019	-3.3	0.83	1.7	-1.5	-13	-0.68	-4	0.74	-2.1	1.5	-1.5
Wits appraisal [mm]	0.26	-4.1	2.3	3	-6.4	-18	-1.3	-5.6	1.7	-2.3	8.6	3
S-N [mm]	67	67	4.4	4.3	42	56	64	64	69	68	81	80
Go-Me [mm]	67	70	5.3	6.2	43	54	63	66	70	72	85	90
NL-ML [°]	24	25	5.8	5.4	5.3	11	20	21	27	28	46	40
NL-NSL [°]	7.3	6.9	3.6	3.1	-3.1	-1.5	5.1	4.9	9.8	9.1	18	16
ML-NSL [°]	31	32	6	6.1	16	14	27	27	35	36	52	48
PFH/AFH [%]	67	66	5.1	5.3	50	54	63	63	71	70	80	84
Gonial_angle [°]	123	125	5.8	6.5	101	103	119	122	126	129	139	142
Facial axis [°]	90	92	4.3	4.7	76	80	88	89	93	95	101	105
NS-Ba [°]	132	130	4.8	5.4	117	110	129	127	135	134	144	144
SN-Pg [°]	79	82	3.3	3.9	67	72	76	79	81	84	88	94
+1/NL [°]	68	65	8.1	7.1	37	46	64	61	73	69	94	85
+1/NSL [°]	76	72	8.5	7.4	43	53	71	67	81	77	98	90
+1/NA [°]	23	27	8	6.7	2.2	10	18	22	28	32	54	44
+1/NA [mm]	3.7	5.1	2.6	2.5	-2.4	-1.3	1.9	3.7	5.3	6.5	11	14
-1/ML[°]	84	90	6.7	7.6	65	68	79	85	88	94	105	111
-1/NB [°]	25	23	6.9	7.4	-1.1	2.5	21	18	30	28	44	42
-1/NB [mm]	3.9	3.1	2.4	2.4	-3.3	-2.8	2.2	1.7	5.6	4.8	12	11
Interincisal angle [°]	128	130	12	12	96	94	120	123	135	137	172	162

Supplementary Table 3. Detailed information about skeletal class I and III patients. Mean, (Std. Dev.)- Standard Deviation, (Min)- Minimum Value, (Pctl. 25)- 25th percentile, (Pctl. 75)- 75th percentile, (Max)-Maximum Value (**2A**). SD = standard deviation, Min = Minimum, Max = Maximum

Supplementary Table 4.

Parameter	Groups	diff	lwr	upr	p adj
SNB [°]	III_Female-I_Female	3.19	2.09	4.29	0.00
SNB [°]	III_Male-I_Female	3.23	2.06	4.41	0.00
SNB [°]	III_Female-I_Male	2.98	1.83	4.14	0.00
SNB [°]	III_Male-I_Male	3.03	1.81	4.25	0.00
SNB [°]	III_0<Age<13-I_0<Age<13	3.17	2.12	4.22	0.00
SNB [°]	III_14<Age<20-I_0<Age<13	3.51	1.70	5.33	0.00
SNB [°]	III_Age>21-I_0<Age<13	4.06	1.73	6.39	0.00
SNB [°]	III_0<Age<13-I_14<Age<20	2.00	0.46	3.55	0.00
SNB [°]	III_14<Age<20-I_14<Age<20	2.35	0.21	4.49	0.02
SNB [°]	III_Age>21-I_14<Age<20	2.90	0.31	5.49	0.02
SNB [°]	III_0<Age<13-I_Age>21	2.85	0.15	5.56	0.03
SNB [°]	III_14<Age<20-I_Age>21	3.19	0.11	6.28	0.04
SNB [°]	III_Age>21-I_Age>21	3.75	0.33	7.16	0.02
SNB [°]	III_Female_0<Age<13-I_Female_0<Age<13	3.36	1.76	4.96	0.00
SNB [°]	III_Female_14<Age<20-I_Female_0<Age<13	3.55	0.35	6.74	0.02
SNB [°]	III_Female_Age>21-I_Female_0<Age<13	3.87	0.26	7.48	0.02
SNB [°]	III_Male_0<Age<13-I_Female_0<Age<13	3.22	1.42	5.02	0.00
SNB [°]	III_Male_14<Age<20-I_Female_0<Age<13	3.72	0.96	6.47	0.00
SNB [°]	III_Male_Age>21-I_Female_0<Age<13	4.61	0.60	8.61	0.01
SNB [°]	III_Female_0<Age<13-I_Male_0<Age<13	3.05	1.37	4.73	0.00
SNB [°]	III_Female_14<Age<20-I_Male_0<Age<13	3.24	0.01	6.47	0.05
SNB [°]	III_Male_0<Age<13-I_Male_0<Age<13	2.92	1.05	4.78	0.00
SNB [°]	III_Male_14<Age<20-I_Male_0<Age<13	3.41	0.61	6.21	0.00
SNB [°]	III_Male_Age>21-I_Male_0<Age<13	4.30	0.26	8.34	0.03
ANB [°]	III_Female-I_Female	-3.20	-3.80	-2.59	0.00
ANB [°]	III_Male-I_Female	-3.60	-4.24	-2.95	0.00
ANB [°]	III_Female-I_Male	-3.12	-3.75	-2.49	0.00
ANB [°]	III_Male-I_Male	-3.52	-4.19	-2.85	0.00
ANB [°]	III_0<Age<13-I_0<Age<13	-3.20	-3.77	-2.62	0.00
ANB [°]	III_14<Age<20-I_0<Age<13	-3.10	-4.09	-2.11	0.00
ANB [°]	III_Age>21-I_0<Age<13	-4.88	-6.15	-3.61	0.00
ANB [°]	III_0<Age<13-I_14<Age<20	-3.20	-4.04	-2.36	0.00
ANB [°]	III_14<Age<20-I_14<Age<20	-3.10	-4.27	-1.94	0.00
ANB [°]	III_Age>21-I_14<Age<20	-4.89	-6.30	-3.47	0.00
ANB [°]	III_0<Age<13-I_Age>21	-2.87	-4.34	-1.39	0.00
ANB [°]	III_14<Age<20-I_Age>21	-2.77	-4.45	-1.08	0.00
ANB [°]	III_Age>21-I_Age>21	-4.55	-6.41	-2.69	0.00
ANB [°]	III_Female_0<Age<13-I_Female_0<Age<13	-3.07	-3.94	-2.20	0.00

ANB [°]	III_Female_14<Age<20-I_Female_0<Age<13	-3.23	-4.97	-1.50	0.00
ANB [°]	III_Female_Age>21-I_Female_0<Age<13	-4.40	-6.36	-2.44	0.00
ANB [°]	III_Male_0<Age<13-I_Female_0<Age<13	-3.51	-4.49	-2.53	0.00
ANB [°]	III_Male_14<Age<20-I_Female_0<Age<13	-3.10	-4.60	-1.61	0.00
ANB [°]	III_Male_Age>21-I_Female_0<Age<13	-5.62	-7.80	-3.44	0.00
ANB [°]	III_Female_0<Age<13-I_Female_14<Age<20	-2.95	-4.24	-1.67	0.00
ANB [°]	III_Female_14<Age<20-I_Female_14<Age<20	-3.11	-5.09	-1.14	0.00
ANB [°]	III_Female_Age>21-I_Female_14<Age<20	-4.28	-6.46	-2.10	0.00
ANB [°]	III_Male_0<Age<13-I_Female_14<Age<20	-3.39	-4.75	-2.03	0.00
ANB [°]	III_Male_14<Age<20-I_Female_14<Age<20	-2.98	-4.75	-1.21	0.00
ANB [°]	III_Male_Age>21-I_Female_14<Age<20	-5.50	-7.87	-3.12	0.00
ANB [°]	III_Female_0<Age<13-I_Female_Age>21	-2.54	-4.79	-0.30	0.01
ANB [°]	III_Female_14<Age<20-I_Female_Age>21	-2.70	-5.41	0.00	0.05
ANB [°]	III_Female_Age>21-I_Female_Age>21	-3.87	-6.72	-1.02	0.00
ANB [°]	III_Male_0<Age<13-I_Female_Age>21	-2.98	-5.27	-0.70	0.00
ANB [°]	III_Male_14<Age<20-I_Female_Age>21	-2.57	-5.13	-0.02	0.05
ANB [°]	III_Male_Age>21-I_Female_Age>21	-5.09	-8.09	-2.08	0.00
ANB [°]	III_Female_0<Age<13-I_Male_0<Age<13	-2.93	-3.84	-2.02	0.00
ANB [°]	III_Female_14<Age<20-I_Male_0<Age<13	-3.09	-4.85	-1.33	0.00
ANB [°]	III_Female_Age>21-I_Male_0<Age<13	-4.26	-6.24	-2.28	0.00
ANB [°]	III_Male_0<Age<13-I_Male_0<Age<13	-3.37	-4.39	-2.36	0.00
ANB [°]	III_Male_14<Age<20-I_Male_0<Age<13	-2.96	-4.48	-1.44	0.00
ANB [°]	III_Male_Age>21-I_Male_0<Age<13	-5.48	-7.67	-3.28	0.00
ANB [°]	III_Female_0<Age<13-I_Male_14<Age<20	-3.10	-4.50	-1.70	0.00
ANB [°]	III_Female_14<Age<20-I_Male_14<Age<20	-3.26	-5.32	-1.21	0.00
ANB [°]	III_Female_Age>21-I_Male_14<Age<20	-4.43	-6.68	-2.18	0.00
ANB [°]	III_Male_0<Age<13-I_Male_14<Age<20	-3.54	-5.02	-2.07	0.00
ANB [°]	III_Male_14<Age<20-I_Male_14<Age<20	-3.13	-4.99	-1.28	0.00
ANB [°]	III_Male_Age>21-I_Male_14<Age<20	-5.65	-8.09	-3.21	0.00
ANB [°]	III_Female_0<Age<13-I_Male_Age>21	-2.86	-5.42	-0.30	0.01
ANB [°]	III_Female_14<Age<20-I_Male_Age>21	-3.03	-5.99	-0.06	0.04
ANB [°]	III_Female_Age>21-I_Male_Age>21	-4.19	-7.30	-1.09	0.00
ANB [°]	III_Male_0<Age<13-I_Male_Age>21	-3.31	-5.90	-0.71	0.00
ANB [°]	III_Male_14<Age<20-I_Male_Age>21	-2.89	-5.73	-0.06	0.04
ANB [°]	III_Male_Age>21-I_Male_Age>21	-5.41	-8.65	-2.16	0.00
Calculated_ANB	III_Female-I_Female	-3.21	-3.60	-2.83	0.00
Calculated_ANB	III_Male-I_Female	-3.49	-3.90	-3.08	0.00
Calculated_ANB	III_Female-I_Male	-3.12	-3.53	-2.72	0.00
Calculated_ANB	III_Male-I_Male	-3.40	-3.83	-2.97	0.00

Calculated_ANB	III_0<Age<13-I_0<Age<13	-3.21	-3.58	-2.84	0.00
Calculated_ANB	III_14<Age<20-I_0<Age<13	-3.13	-3.76	-2.50	0.00
Calculated_ANB	III_Age>21-I_0<Age<13	-4.49	-5.30	-3.68	0.00
Calculated_ANB	III_0<Age<13-I_14<Age<20	-3.10	-3.63	-2.56	0.00
Calculated_ANB	III_14<Age<20-I_14<Age<20	-3.01	-3.76	-2.27	0.00
Calculated_ANB	III_Age>21-I_14<Age<20	-4.38	-5.28	-3.48	0.00
Calculated_ANB	III_0<Age<13-I_Age>21	-2.88	-3.83	-1.94	0.00
Calculated_ANB	III_14<Age<20-I_Age>21	-2.80	-3.88	-1.73	0.00
Calculated_ANB	III_Age>21-I_Age>21	-4.17	-5.36	-2.98	0.00
Calculated_ANB	III_Female_0<Age<13-I_Female_0<Age<13	-3.20	-3.75	-2.64	0.00
Calculated_ANB	III_Female_14<Age<20-I_Female_0<Age<13	-2.83	-3.93	-1.72	0.00
Calculated_ANB	III_Female_Age>21-I_Female_0<Age<13	-4.42	-5.67	-3.17	0.00
Calculated_ANB	III_Male_0<Age<13-I_Female_0<Age<13	-3.40	-4.02	-2.77	0.00
Calculated_ANB	III_Male_14<Age<20-I_Female_0<Age<13	-3.47	-4.42	-2.51	0.00
Calculated_ANB	III_Male_Age>21-I_Female_0<Age<13	-4.75	-6.14	-3.36	0.00
Calculated_ANB	III_Female_0<Age<13-I_Female_14<Age<20	-2.89	-3.70	-2.07	0.00
Calculated_ANB	III_Female_14<Age<20-I_Female_14<Age<20	-2.52	-3.77	-1.26	0.00
Calculated_ANB	III_Female_Age>21-I_Female_14<Age<20	-4.11	-5.49	-2.72	0.00
Calculated_ANB	III_Male_0<Age<13-I_Female_14<Age<20	-3.08	-3.95	-2.22	0.00
Calculated_ANB	III_Male_14<Age<20-I_Female_14<Age<20	-3.16	-4.28	-2.03	0.00
Calculated_ANB	III_Male_Age>21-I_Female_14<Age<20	-4.44	-5.95	-2.92	0.00
Calculated_ANB	III_Female_0<Age<13-I_Female_Age>21	-2.86	-4.29	-1.44	0.00
Calculated_ANB	III_Female_14<Age<20-I_Female_Age>21	-2.49	-4.21	-0.78	0.00
Calculated_ANB	III_Female_Age>21-I_Female_Age>21	-4.09	-5.90	-2.27	0.00
Calculated_ANB	III_Male_0<Age<13-I_Female_Age>21	-3.06	-4.52	-1.61	0.00
Calculated_ANB	III_Male_14<Age<20-I_Female_Age>21	-3.13	-4.76	-1.51	0.00
Calculated_ANB	III_Male_Age>21-I_Female_Age>21	-4.42	-6.33	-2.50	0.00
Calculated_ANB	III_Female_0<Age<13-I_Male_0<Age<13	-3.03	-3.61	-2.45	0.00
Calculated_ANB	III_Female_14<Age<20-I_Male_0<Age<13	-2.66	-3.78	-1.54	0.00
Calculated_ANB	III_Female_Age>21-I_Male_0<Age<13	-4.25	-5.51	-2.99	0.00
Calculated_ANB	III_Male_0<Age<13-I_Male_0<Age<13	-3.23	-3.88	-2.58	0.00
Calculated_ANB	III_Male_14<Age<20-I_Male_0<Age<13	-3.30	-4.27	-2.33	0.00
Calculated_ANB	III_Male_Age>21-I_Male_0<Age<13	-4.58	-5.98	-3.18	0.00
Calculated_ANB	III_Female_0<Age<13-I_Male_14<Age<20	-3.18	-4.07	-2.28	0.00
Calculated_ANB	III_Female_14<Age<20-I_Male_14<Age<20	-2.81	-4.11	-1.50	0.00
Calculated_ANB	III_Female_Age>21-I_Male_14<Age<20	-4.40	-5.83	-2.97	0.00
Calculated_ANB	III_Male_0<Age<13-I_Male_14<Age<20	-3.37	-4.31	-2.44	0.00
Calculated_ANB	III_Male_14<Age<20-I_Male_14<Age<20	-3.45	-4.63	-2.26	0.00
Calculated_ANB	III_Male_Age>21-I_Male_14<Age<20	-4.73	-6.28	-3.17	0.00

Calculated_ANB	III_Female_0<Age<13-I_Male_Age>21	-2.72	-4.35	-1.09	0.00
Calculated_ANB	III_Female_14<Age<20-I_Male_Age>21	-2.35	-4.24	-0.46	0.00
Calculated_ANB	III_Female_Age>21-I_Male_Age>21	-3.94	-5.91	-1.96	0.00
Calculated_ANB	III_Male_0<Age<13-I_Male_Age>21	-2.92	-4.57	-1.26	0.00
Calculated_ANB	III_Male_14<Age<20-I_Male_Age>21	-2.99	-4.79	-1.18	0.00
Calculated_ANB	III_Male_Age>21-I_Male_Age>21	-4.27	-6.33	-2.20	0.00
SN-Pg [°]	III_Female-I_Female	3.36	2.22	4.50	0.00
SN-Pg [°]	III_Male-I_Female	3.36	2.14	4.58	0.00
SN-Pg [°]	III_Female-I_Male	3.03	1.84	4.23	0.00
SN-Pg [°]	III_Male-I_Male	3.03	1.76	4.30	0.00
SN-Pg [°]	III_0<Age<13-I_0<Age<13	3.15	2.06	4.24	0.00
SN-Pg [°]	III_14<Age<20-I_0<Age<13	3.79	1.91	5.67	0.00
SN-Pg [°]	III_Age>21-I_0<Age<13	4.97	2.56	7.38	0.00
SN-Pg [°]	III_0<Age<13-I_14<Age<20	1.82	0.23	3.42	0.01
SN-Pg [°]	III_14<Age<20-I_14<Age<20	2.46	0.25	4.67	0.02
SN-Pg [°]	III_Age>21-I_14<Age<20	3.64	0.97	6.32	0.00
SN-Pg [°]	III_14<Age<20-I_Age>21	3.22	0.03	6.41	0.05
SN-Pg [°]	III_Age>21-I_Age>21	4.40	0.87	7.93	0.01
SN-Pg [°]	III_Female_0<Age<13-I_Female_0<Age<13	3.45	1.80	5.11	0.00
SN-Pg [°]	III_Female_14<Age<20-I_Female_0<Age<13	4.11	0.82	7.41	0.00
SN-Pg [°]	III_Female_Age>21-I_Female_0<Age<13	4.59	0.86	8.31	0.00
SN-Pg [°]	III_Male_0<Age<13-I_Female_0<Age<13	3.22	1.37	5.08	0.00
SN-Pg [°]	III_Male_14<Age<20-I_Female_0<Age<13	3.91	1.06	6.75	0.00
SN-Pg [°]	III_Male_Age>21-I_Female_0<Age<13	5.91	1.77	10.05	0.00
SN-Pg [°]	III_Female_0<Age<13-I_Male_0<Age<13	2.98	1.25	4.71	0.00
SN-Pg [°]	III_Female_14<Age<20-I_Male_0<Age<13	3.64	0.31	6.98	0.02
SN-Pg [°]	III_Female_Age>21-I_Male_0<Age<13	4.12	0.36	7.88	0.02
SN-Pg [°]	III_Male_0<Age<13-I_Male_0<Age<13	2.75	0.83	4.68	0.00
SN-Pg [°]	III_Male_14<Age<20-I_Male_0<Age<13	3.44	0.55	6.33	0.01
SN-Pg [°]	III_Male_Age>21-I_Male_0<Age<13	5.44	1.27	9.61	0.00
Wits appraisal [mm]	III_Female-I_Female	-4.09	-4.90	-3.27	0.00
Wits appraisal [mm]	III_Male-I_Female	-4.19	-5.07	-3.32	0.00
Wits appraisal [mm]	III_Female-I_Male	-4.71	-5.56	-3.85	0.00
Wits appraisal [mm]	III_Male-I_Male	-4.81	-5.73	-3.90	0.00
Wits appraisal [mm]	III_0<Age<13-I_0<Age<13	-4.14	-4.94	-3.35	0.00
Wits appraisal [mm]	III_14<Age<20-I_0<Age<13	-4.51	-5.87	-3.15	0.00
Wits appraisal [mm]	III_Age>21-I_0<Age<13	-4.83	-6.58	-3.08	0.00
Wits appraisal [mm]	III_0<Age<13-I_14<Age<20	-4.82	-5.98	-3.66	0.00
Wits appraisal [mm]	III_14<Age<20-I_14<Age<20	-5.18	-6.79	-3.58	0.00
Wits appraisal [mm]	III_Age>21-I_14<Age<20	-5.50	-7.44	-3.56	0.00
Wits appraisal [mm]	III_0<Age<13-I_Age>21	-4.30	-6.33	-2.27	0.00

Wits appraisal [mm]	III_14<Age<20-I_Age>21	-4.66	-6.98	-2.35	0.00
Wits appraisal [mm]	III_Age>21-I_Age>21	-4.98	-7.54	-2.42	0.00
Wits appraisal [mm]	III_Female_0<Age<13-I_Female_0<Age<13	-3.92	-5.11	-2.72	0.00
Wits appraisal [mm]	III_Female_14<Age<20-I_Female_0<Age<13	-4.22	-6.61	-1.84	0.00
Wits appraisal [mm]	III_Female_Age>21-I_Female_0<Age<13	-4.29	-6.98	-1.60	0.00
Wits appraisal [mm]	III_Male_0<Age<13-I_Female_0<Age<13	-3.90	-5.24	-2.55	0.00
Wits appraisal [mm]	III_Male_14<Age<20-I_Female_0<Age<13	-4.31	-6.36	-2.25	0.00
Wits appraisal [mm]	III_Male_Age>21-I_Female_0<Age<13	-4.96	-7.96	-1.97	0.00
Wits appraisal [mm]	III_Female_0<Age<13-I_Female_14<Age<20	-4.40	-6.16	-2.63	0.00
Wits appraisal [mm]	III_Female_14<Age<20-I_Female_14<Age<20	-4.70	-7.42	-1.99	0.00
Wits appraisal [mm]	III_Female_Age>21-I_Female_14<Age<20	-4.77	-7.76	-1.78	0.00
Wits appraisal [mm]	III_Male_0<Age<13-I_Female_14<Age<20	-4.38	-6.25	-2.51	0.00
Wits appraisal [mm]	III_Male_14<Age<20-I_Female_14<Age<20	-4.79	-7.22	-2.35	0.00
Wits appraisal [mm]	III_Male_Age>21-I_Female_14<Age<20	-5.44	-8.71	-2.18	0.00
Wits appraisal [mm]	III_Female_0<Age<13-I_Female_Age>21	-4.08	-7.16	-0.99	0.00
Wits appraisal [mm]	III_Female_14<Age<20-I_Female_Age>21	-4.39	-8.09	-0.68	0.01
Wits appraisal [mm]	III_Female_Age>21-I_Female_Age>21	-4.45	-8.37	-0.54	0.01
Wits appraisal [mm]	III_Male_0<Age<13-I_Female_Age>21	-4.06	-7.20	-0.92	0.00
Wits appraisal [mm]	III_Male_14<Age<20-I_Female_Age>21	-4.47	-7.98	-0.96	0.00
Wits appraisal [mm]	III_Male_Age>21-I_Female_Age>21	-5.13	-9.25	-1.00	0.00
Wits appraisal [mm]	III_Female_0<Age<13-I_Male_0<Age<13	-4.46	-5.71	-3.21	0.00
Wits appraisal [mm]	III_Female_14<Age<20-I_Male_0<Age<13	-4.77	-7.18	-2.36	0.00
Wits appraisal [mm]	III_Female_Age>21-I_Male_0<Age<13	-4.84	-7.56	-2.12	0.00
Wits appraisal [mm]	III_Male_0<Age<13-I_Male_0<Age<13	-4.45	-5.84	-3.05	0.00
Wits appraisal [mm]	III_Male_14<Age<20-I_Male_0<Age<13	-4.85	-6.94	-2.76	0.00
Wits appraisal [mm]	III_Male_Age>21-I_Male_0<Age<13	-5.51	-8.53	-2.49	0.00
Wits appraisal [mm]	III_Female_0<Age<13-I_Male_14<Age<20	-5.37	-7.30	-3.45	0.00
Wits appraisal [mm]	III_Female_14<Age<20-I_Male_14<Age<20	-5.68	-8.51	-2.86	0.00
Wits appraisal [mm]	III_Female_Age>21-I_Male_14<Age<20	-5.75	-8.84	-2.66	0.00
Wits appraisal [mm]	III_Male_0<Age<13-I_Male_14<Age<20	-5.36	-7.38	-3.34	0.00
Wits appraisal [mm]	III_Male_14<Age<20-I_Male_14<Age<20	-5.77	-8.32	-3.21	0.00
Wits appraisal [mm]	III_Male_Age>21-I_Male_14<Age<20	-6.42	-9.78	-3.07	0.00
Wits appraisal [mm]	III_Female_0<Age<13-I_Male_Age>21	-4.61	-8.13	-1.10	0.00
Wits appraisal [mm]	III_Female_14<Age<20-I_Male_Age>21	-4.92	-9.00	-0.85	0.00
Wits appraisal [mm]	III_Female_Age>21-I_Male_Age>21	-4.99	-9.25	-0.73	0.01
Wits appraisal [mm]	III_Male_0<Age<13-I_Male_Age>21	-4.60	-8.16	-1.03	0.00
Wits appraisal [mm]	III_Male_14<Age<20-I_Male_Age>21	-5.01	-8.90	-1.11	0.00
Wits appraisal [mm]	III_Male_Age>21-I_Male_Age>21	-5.66	-10.12	-1.20	0.00
S-N (mm)	III_Male-I_Female	1.70	0.22	3.19	0.02

S-N (mm)	III_Female-I_Male	-2.15	-3.61	-0.70	0.00
S-N (mm)	III_14<Age<20-I_0<Age<13	2.46	0.14	4.77	0.03
S-N (mm)	III_0<Age<13-I_14<Age<20	-2.55	-4.51	-0.58	0.00
S-N (mm)	III_Female_0<Age<13-I_Male_0<Age<13	-2.70	-4.77	-0.63	0.00
S-N (mm)	III_Female_0<Age<13-I_Male_14<Age<20	-5.18	-8.36	-2.00	0.00
Go-Me (mm)	III_Female-I_Female	2.77	0.95	4.58	0.00
Go-Me (mm)	III_Male-I_Female	4.49	2.55	6.43	0.00
Go-Me (mm)	III_Male-I_Male	2.62	0.60	4.64	0.00
Go-Me (mm)	III_0<Age<13-I_0<Age<13	1.89	0.25	3.53	0.01
Go-Me (mm)	III_14<Age<20-I_0<Age<13	7.19	4.36	10.01	0.00
Go-Me (mm)	III_Age>21-I_0<Age<13	8.77	5.14	12.39	0.00
Go-Me (mm)	III_0<Age<13-I_14<Age<20	-2.98	-5.38	-0.58	0.01
Go-Me (mm)	III_14<Age<20-I_Age>21	5.83	1.03	10.63	0.01
Go-Me (mm)	III_Age>21-I_Age>21	7.41	2.10	12.72	0.00
Go-Me (mm)	III_Female_14<Age<20-I_Female_0<Age<13	7.54	2.67	12.40	0.00
Go-Me (mm)	III_Female_Age>21-I_Female_0<Age<13	7.44	1.94	12.94	0.00
Go-Me (mm)	III_Male_0<Age<13-I_Female_0<Age<13	3.20	0.46	5.95	0.01
Go-Me (mm)	III_Male_14<Age<20-I_Female_0<Age<13	8.38	4.19	12.58	0.00
Go-Me (mm)	III_Male_Age>21-I_Female_0<Age<13	12.31	6.20	18.42	0.00
Go-Me (mm)	III_Male_Age>21-I_Female_14<Age<20	7.90	1.24	14.56	0.01
Go-Me (mm)	III_Female_14<Age<20-I_Male_0<Age<13	5.60	0.67	10.52	0.01
Go-Me (mm)	III_Male_14<Age<20-I_Male_0<Age<13	6.44	2.18	10.71	0.00
Go-Me (mm)	III_Male_Age>21-I_Male_0<Age<13	10.37	4.22	16.53	0.00
Go-Me (mm)	III_Female_0<Age<13-I_Male_14<Age<20	-4.98	-8.91	-1.05	0.00
Go-Me (mm)	III_Male_0<Age<13-I_Male_14<Age<20	-4.17	-8.29	-0.04	0.05
Go-Me (mm)	III_Male_14<Age<20-I_Male_Age>21	8.59	0.65	16.54	0.02
Go-Me (mm)	III_Male_Age>21-I_Male_Age>21	12.53	3.43	21.62	0.00
NL/ML angle	III_0<Age<13-I_14<Age<20	2.75	0.16	5.34	0.03
PFH/AFH ratio	III_0<Age<13-I_14<Age<20	-2.68	-5.04	-0.32	0.02
Gonial angle [°]	III_Female-I_Female	2.24	0.26	4.22	0.02
Gonial angle [°]	III_Male-I_Female	3.25	1.13	5.36	0.00
Gonial angle [°]	III_Male-I_Male	2.87	0.66	5.07	0.00
Gonial angle [°]	III_0<Age<13-I_0<Age<13	2.41	0.52	4.31	0.00
Gonial angle [°]	III_0<Age<13-I_14<Age<20	5.14	2.37	7.91	0.00
Gonial angle [°]	III_Male_0<Age<13-I_Female_0<Age<13	3.68	0.48	6.89	0.01
Gonial angle [°]	III_Male_0<Age<13-I_Female_14<Age<20	5.70	1.24	10.15	0.00
Gonial angle [°]	III_Female_0<Age<13-I_Male_14<Age<20	5.02	0.43	9.61	0.02
Gonial angle [°]	III_Male_0<Age<13-I_Male_14<Age<20	6.86	2.04	11.68	0.00
Facial Axis [°]	III_Female-I_Female	2.35	0.89	3.82	0.00
Facial Axis [°]	III_Female-I_Male	2.04	0.51	3.58	0.00
Facial Axis [°]	III_0<Age<13-I_0<Age<13	1.85	0.44	3.27	0.00

Facial Axis [°]	III_Female_0<Age<13-I_Female_0<Age<13	2.23	0.09	4.36	0.03
Facial Axis [°]	III_Female_14<Age<20-I_Female_0<Age<13	4.33	0.08	8.58	0.04
+1/NL [°]	III_Female-I_Female	-3.17	-5.70	-0.64	0.01
+1/NL [°]	III_Male-I_Female	-4.42	-7.12	-1.71	0.00
+1/NL [°]	III_Male-I_Male	-3.10	-5.91	-0.28	0.02
+1/NL [°]	III_0<Age<13-I_0<Age<13	-3.43	-5.86	-0.99	0.00
+1/NL [°]	III_0<Age<13-I_14<Age<20	-5.18	-8.74	-1.62	0.00
+1/NL [°]	III_Male_0<Age<13-I_Female_0<Age<13	-4.28	-8.41	-0.15	0.03
+1/NL [°]	III_Female_0<Age<13-I_Female_14<Age<20	-5.67	-11.09	-0.25	0.03
+1/NL [°]	III_Male_0<Age<13-I_Female_14<Age<20	-6.42	-12.16	-0.69	0.01
+1/NSL [°]	III_Female-I_Female	-3.58	-6.22	-0.94	0.00
+1/NSL [°]	III_Male-I_Female	-5.47	-8.30	-2.65	0.00
+1/NSL [°]	III_Male-I_Male	-3.52	-6.46	-0.57	0.01
+1/NSL [°]	III_0<Age<13-I_0<Age<13	-3.77	-6.32	-1.22	0.00
+1/NSL [°]	III_0<Age<13-I_14<Age<20	-6.06	-9.80	-2.33	0.00
+1/NSL [°]	III_Female_0<Age<13-I_Female_0<Age<13	-3.98	-7.82	-0.13	0.04
+1/NSL [°]	III_Male_0<Age<13-I_Female_0<Age<13	-5.62	-9.94	-1.29	0.00
+1/NSL [°]	III_Female_0<Age<13-I_Female_14<Age<20	-5.78	-11.46	-0.11	0.04
+1/NSL [°]	III_Male_0<Age<13-I_Female_14<Age<20	-7.43	-13.43	-1.42	0.00
+1/NA [°]	III_Female-I_Female	3.58	1.12	6.05	0.00
+1/NA [°]	III_Male-I_Female	5.84	3.21	8.48	0.00
+1/NA [°]	III_Male-I_Male	4.02	1.27	6.76	0.00
+1/NA [°]	III_0<Age<13-I_0<Age<13	3.80	1.43	6.17	0.00
+1/NA [°]	III_0<Age<13-I_14<Age<20	7.26	3.80	10.73	0.00
+1/NA [°]	III_14<Age<20-I_14<Age<20	5.43	0.62	10.24	0.02
+1/NA [°]	III_Age>21-I_14<Age<20	5.92	0.09	11.74	0.04
+1/NA [°]	III_Female_0<Age<13-I_Female_0<Age<13	3.68	0.11	7.25	0.04
+1/NA [°]	III_Male_0<Age<13-I_Female_0<Age<13	5.91	1.91	9.92	0.00
+1/NA [°]	III_Female_0<Age<13-I_Female_14<Age<20	6.78	1.51	12.04	0.00
+1/NA [°]	III_Male_0<Age<13-I_Female_14<Age<20	9.01	3.44	14.58	0.00
+1/NA [°]	III_Male_14<Age<20-I_Female_14<Age<20	7.64	0.39	14.89	0.03
+1/NA [°]	III_Female_0<Age<13-I_Male_14<Age<20	5.75	0.00	11.49	0.05
+1/NA [°]	III_Male_0<Age<13-I_Male_14<Age<20	7.98	1.95	14.01	0.00
+1/NA [mm]	III_Female-I_Female	1.18	0.34	2.01	0.00
+1/NA [mm]	III_Male-I_Female	2.03	1.14	2.92	0.00
+1/NA [mm]	III_Male-I_Male	1.58	0.65	2.51	0.00
+1/NA [mm]	III_0<Age<13-I_0<Age<13	1.17	0.36	1.97	0.00
+1/NA [mm]	III_14<Age<20-I_0<Age<13	1.62	0.23	3.00	0.01
+1/NA [mm]	III_Age>21-I_0<Age<13	2.08	0.30	3.86	0.01

+1/NA [mm]	III_0<Age<13-I_14<Age<20	1.63	0.45	2.81	0.00
+1/NA [mm]	III_14<Age<20-I_14<Age<20	2.08	0.44	3.71	0.00
+1/NA [mm]	III_Age>21-I_14<Age<20	2.54	0.56	4.52	0.00
+1/NA [mm]	III_Male_0<Age<13-I_Female_0<Age<13	1.92	0.55	3.28	0.00
+1/NA [mm]	III_Male_14<Age<20-I_Female_0<Age<13	2.41	0.33	4.50	0.01
+1/NA [mm]	III_Male_0<Age<13-I_Female_14<Age<20	2.20	0.30	4.09	0.01
+1/NA [mm]	III_Male_14<Age<20-I_Female_14<Age<20	2.69	0.23	5.16	0.02
+1/NA [mm]	III_Male_0<Age<13-I_Male_14<Age<20	2.08	0.03	4.13	0.04
-1/ML [°] (anatomic)	III_Female-I_Female	5.83	3.53	8.13	0.00
-1/ML [°] (anatomic)	III_Male-I_Female	5.97	3.51	8.42	0.00
-1/ML [°] (anatomic)	III_Female-I_Male	5.48	3.07	7.89	0.00
-1/ML [°] (anatomic)	III_Male-I_Male	5.62	3.06	8.17	0.00
-1/ML [°] (anatomic)	III_0<Age<13-I_0<Age<13	5.43	3.22	7.64	0.00
-1/ML [°] (anatomic)	III_14<Age<20-I_0<Age<13	4.74	0.93	8.55	0.01
-1/ML [°] (anatomic)	III_Age>21-I_0<Age<13	7.24	2.35	12.14	0.00
-1/ML [°] (anatomic)	III_0<Age<13-I_14<Age<20	6.47	3.23	9.70	0.00
-1/ML [°] (anatomic)	III_14<Age<20-I_14<Age<20	5.77	1.29	10.26	0.00
-1/ML [°] (anatomic)	III_Age>21-I_14<Age<20	8.28	2.85	13.72	0.00
-1/ML [°] (anatomic)	III_0<Age<13-I_Age>21	7.17	1.49	12.85	0.00
-1/ML [°] (anatomic)	III_14<Age<20-I_Age>21	6.48	0.01	12.95	0.05
-1/ML [°] (anatomic)	III_Age>21-I_Age>21	8.99	1.83	16.15	0.00
-1/ML [°] (anatomic)	III_Female_0<Age<13-I_Female_0<Age<13	5.60	2.24	8.97	0.00
-1/ML [°] (anatomic)	III_Male_0<Age<13-I_Female_0<Age<13	5.60	1.82	9.38	0.00
-1/ML [°] (anatomic)	III_Female_0<Age<13-I_Female_14<Age<20	6.24	1.28	11.20	0.00
-1/ML [°] (anatomic)	III_Male_0<Age<13-I_Female_14<Age<20	6.24	0.99	11.49	0.01
-1/ML [°] (anatomic)	III_Female_0<Age<13-I_Male_0<Age<13	5.20	1.68	8.72	0.00
-1/ML [°] (anatomic)	III_Male_0<Age<13-I_Male_0<Age<13	5.20	1.28	9.12	0.00
-1/ML [°] (anatomic)	III_Female_0<Age<13-I_Male_14<Age<20	6.76	1.34	12.17	0.00
-1/ML [°] (anatomic)	III_Male_0<Age<13-I_Male_14<Age<20	6.75	1.07	12.43	0.01
-1/NB [°]	III_Female-I_Female	-2.54	-4.86	-0.23	0.02
-1/NB [°]	III_Male-I_Female	-2.53	-5.00	-0.05	0.04
-1/NB [mm]	III_Female-I_Female	-0.90	-1.68	-0.12	0.02
-1/NB [mm]	III_Female-I_Male	-1.04	-1.86	-0.22	0.01
-1/NB [mm]	III_0<Age<13-I_0<Age<13	-0.87	-1.62	-0.12	0.01
-1/NB [mm]	III_0<Age<13-I_14<Age<20	-1.24	-2.35	-0.14	0.02

Supplementary Table 4. Multiple groups comparisons between skeletal class I and III groups, using the Tukey method. The table represents the cephalometric parameter, Groups of comparisons, the difference between the groups (diff), the lower (lwr), and (upr) limits, and the adjusted tukey p value (p adj). diff = difference, lwr = lower limit, upr = upper limit, p adj = Tukey adjusted p-value

Supplementary Figure 1.

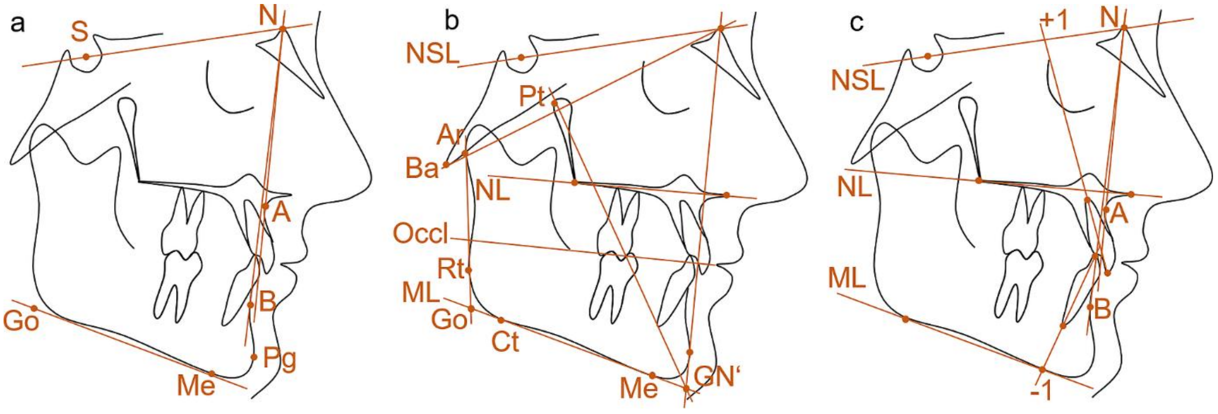

Supplementary Figure 1. Representation of the important landmarks, as presented in figures 1a-c.

Supplementary Figure 2.

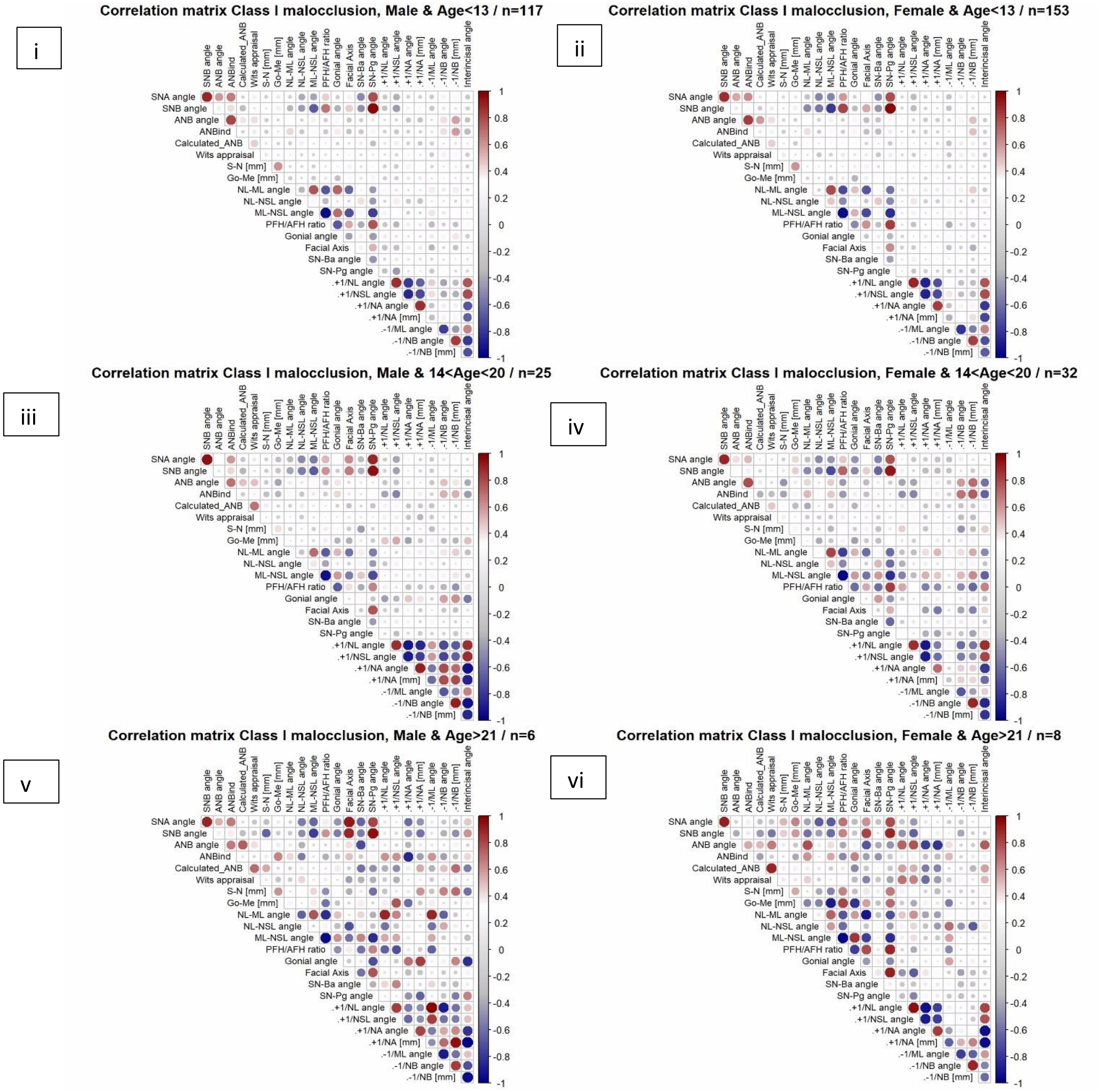

Supplementary Figure 2(i-vi). These heatmaps present the Spearman correlation between different cephalometric parameters for skeletal class I patients. Colour coding signifies the strength and direction of the correlation: blue indicates a negative correlation, while red indicates a positive correlation, and the intensity of the color reflects the correlation strength. **Supplementary** Figures 2i - vi present correlations for skeletal class I patients, grouped by the different gender and age subgroups.

Supplementary Figure 3.

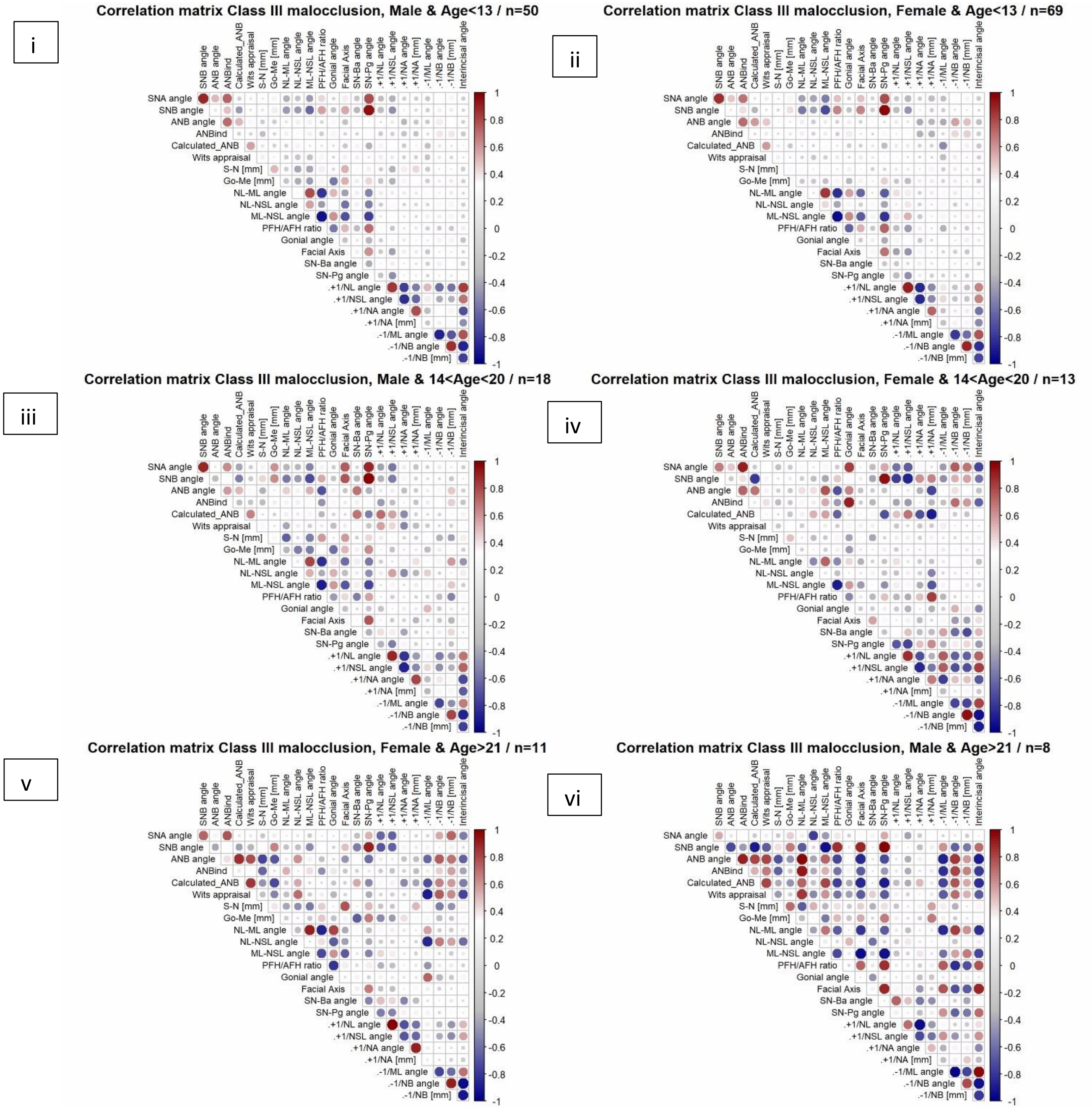

Supplementary Figure 3(i-vi). These heatmaps present the Spearman correlation between different cephalometric parameters for skeletal class III patients. Colour coding signifies the strength and direction of the correlation: blue indicates a negative correlation, while red indicates a positive correlation, and the intensity of the color reflects the correlation strength. **Supplementary** Figures 3i - vi present correlations for skeletal class III patients, grouped by the different gender and age subgroups.
